# Systems Analysis of a RIG-I Agonist Inducing Broad Spectrum Inhibition of Virus Infectivity

**DOI:** 10.1371/journal.ppat.1003298

**Published:** 2013-04-25

**Authors:** Marie-Line Goulet, David Olagnier, Zhengyun Xu, Suzanne Paz, S. Mehdi Belgnaoui, Erin I. Lafferty, Valérie Janelle, Meztli Arguello, Marilene Paquet, Khader Ghneim, Stephanie Richards, Andrew Smith, Peter Wilkinson, Mark Cameron, Ulrich Kalinke, Salman Qureshi, Alain Lamarre, Elias K. Haddad, Rafick Pierre Sekaly, Suraj Peri, Siddharth Balachandran, Rongtuan Lin, John Hiscott

**Affiliations:** 1 Lady Davis Institute, Jewish General Hospital, McGill University, Montréal, Canada; 2 Division of Infectious Diseases, Vaccine & Gene Therapy Institute of Florida, Port Saint Lucie, Florida, United States of America; 3 Division of Experimental Medicine, McGill University, Montréal, Canada; 4 Immunovirology Laboratory, INRS-Institut Armand-Frappier, Laval, Quebec, Canada; 5 Comparative Medicine & Animal Resources Centre, McGill University, Montréal, Canada; 6 Institute for Experimental Infection Research, TWINCORE, Hannover, Germany; 7 Fox Chase Cancer Center, Philadelphia, Pennsylvania, United States of America; Cleveland Clinic, United States of America

## Abstract

The RIG-I like receptor pathway is stimulated during RNA virus infection by interaction between cytosolic RIG-I and viral RNA structures that contain short hairpin dsRNA and 5′ triphosphate (5′ppp) terminal structure. In the present study, an RNA agonist of RIG-I was synthesized *in vitro* and shown to stimulate RIG-I-dependent antiviral responses at concentrations in the picomolar range. In human lung epithelial A549 cells, 5′pppRNA specifically stimulated multiple parameters of the innate antiviral response, including IRF3, IRF7 and STAT1 activation, and induction of inflammatory and interferon stimulated genes - hallmarks of a fully functional antiviral response. Evaluation of the magnitude and duration of gene expression by transcriptional profiling identified a robust, sustained and diversified antiviral and inflammatory response characterized by enhanced pathogen recognition and interferon (IFN) signaling. Bioinformatics analysis further identified a transcriptional signature uniquely induced by 5′pppRNA, and not by IFNα-2b, that included a constellation of IRF7 and NF-kB target genes capable of mobilizing multiple arms of the innate and adaptive immune response. Treatment of primary PBMCs or lung epithelial A549 cells with 5′pppRNA provided significant protection against a spectrum of RNA and DNA viruses. In C57Bl/6 mice, intravenous administration of 5′pppRNA protected animals from a lethal challenge with H1N1 Influenza, reduced virus titers in mouse lungs and protected animals from virus-induced pneumonia. Strikingly, the RIG-I-specific transcriptional response afforded partial protection from influenza challenge, even in the absence of type I interferon signaling. This systems approach provides transcriptional, biochemical, and *in vivo* analysis of the antiviral efficacy of 5′pppRNA and highlights the therapeutic potential associated with the use of RIG-I agonists as broad spectrum antiviral agents.

## Author Summary

Development of safe and effective drugs that inhibit virus replication remains a challenge. Activation of natural host defense using interferon (IFN) therapy has proven an effective treatment of certain viral infections. As a distinct variation on this concept, we analyzed the capacity of small RNA molecules that mimic viral components to trigger the host antiviral response and to inhibit the replication of several pathogenic human viruses. Using gene expression profiling, we identified robust antiviral and inflammatory gene signatures after treatment with a 5′-triphosphate containing RNA (5′pppRNA), including an integrated set of genes that is not regulated by IFN treatment. Delivery of 5′pppRNA into lung epithelial cells *in vitro* stimulated a strong antiviral immune response that inhibited the multiplication of several viruses. In a murine model of influenza infection, inoculation of the agonist protected animals from a lethal challenge of H1N1 influenza and inhibited virus replication in mouse lungs during the first 24–48 h after infection. This report highlights the therapeutic potential of naturally derived RIG-I agonists as potent stimulators of the innate antiviral response, with the capacity to block the replication of diverse human pathogenic viruses.

## Introduction

The innate immune system has evolved numerous molecular sensors and signaling pathways to detect, contain and clear viral infections [1]–[4]. Viruses are sensed by a subset of pattern recognition receptors (PRRs) that recognize evolutionarily conserved structures known as pathogen-associated molecular patterns (PAMPs). Classically, viral nucleic acids are the predominant PAMPs detected by these receptors during infection. These sensing steps contribute to the activation of signaling cascades that culminate in the early production of antiviral effector molecules, cytokines and chemokines responsible for the inhibition of viral replication and the induction of adaptive immune responses [2], [5]–[7]. In addition to the nucleic acid sensing by a subset of endosome-associated Toll-like receptors (TLR), viral RNA structures within the cytoplasm are recognized by members of the retinoic acid-inducible gene-I (RIG-I)-like receptors (RLRs) family, including the three DExD/H box RNA helicases RIG-I, Mda5 and LGP-2 [2], [3], [5], [8]–[12].

RIG-I is a cytosolic multidomain protein that detects viral RNA through its helicase domain [13], [14]. In addition to its RNA sensing domain, RIG-I also possesses an effector caspase activation and recruitment domain (CARD) that interacts with the mitochondrial adaptor MAVS, also known as VISA, IPS-1, Cardif [15], [16]. Viral RNA binding alters RIG-I conformation from an auto-inhibitory state to an open conformation exposing the CARD domain, resulting in the generation of an activated state characterized by ATP hydrolysis and ATP-driven translocation on RNA [17]–[19]. Activation of RIG-I also allows ubiquitination and/or binding to polyubiquitin. In recent studies, polyubiquitin binding has been shown to induce formation of RIG-I tetramers that activate downstream signaling by inducing the formation of prion-like fibrils composed of the MAVS adaptor [20]. MAVS then triggers the activation of IRF3 and NF-κB transcription proteins through the IKK-related serine kinases TBK1 and IKKε [10], [21]–[23], leading to the primary activation of the antiviral program, involving production of type I interferons (IFNβ and IFNα), as well as pro-inflammatory cytokines and antiviral factors [5], [24], [25]. A secondary response involving the induction of IFN stimulated genes (ISGs) is induced by the binding of IFN to its cognate receptor (IFNα/βR), which triggers the JAK-STAT pathway to amplify the antiviral immune response [6], [26]–[29].

The nature of the ligand recognized by RIG-I has been the subject of intense study given that these PAMPs are the initial triggers of the antiviral immune response. *In vitro* synthesized RNA carrying an exposed 5′ terminal triphosphate (5′ppp) moiety was first identified as RIG-I agonists [30]–[32]. The 5′ppp moiety is present at the end of viral and self RNA molecules generated by RNA polymerization; however, in eukaryotic cells, RNA processing in the nucleus cleaves the 5′ppp end and the RNA is capped prior to release into the cytoplasm. This mechanism distinguishes viral ‘non-self’ 5′pppRNA from cellular ‘self’ RNA, and renders it recognizable to the innate RIG-I sensor [30], [31], [33]. Further characterization of the RNA structure demonstrated that blunt base pairing at the 5′ end of the RNA, with a minimum double strand (ds) length of 20 nucleotides was also important for RIG-I signaling [17], [33], [34]. Furthermore, short dsRNA (<300 bp) triggered RIG-I, whereas long dsRNA (>2000 bp) such as poly I:C and lacking 5′ppp failed to trigger RIG-I, but was recognized by Mda5 [35].

Natural RNA extracted from virally infected cells, specifically the viral RNA genome or viral replicative intermediates, were also shown to activate RIG-I [30], [31], [36]–[38]. Interestingly, the highly conserved 5′ and 3′ untranslated regions (UTRs) of negative single strand RNA virus genomes display high base pair complementarity and the panhandle structure theoretically formed by the viral genome meets the requirements for RIG-I recognition [17]. The elucidation of the crystal structure of RIG-I highlighted the molecular interactions between RIG-I and 5′ppp dsRNA [18], [39], providing a structural basis for the conformational changes involved in exposing the CARD domain for effective downstream signaling [18].

Given the level of molecular understanding of the RIG-I ligand and subsequent signaling leading to induction of antiviral immune response, we sought to investigate the range of the protective innate immune response triggered by RIG-I agonists against viral infections. A short *in vitro*-synthesized 5′pppRNA derived from the 5′ and 3′ UTRs of the VSV genome activated the RIG-I signaling pathway and triggered a robust antiviral response that interfered with infection by several pathogenic viruses, including Dengue, HCV, H1N1 Influenza A/PR/8/34 and HIV-1. Furthermore, intravenous delivery of the RNA agonist stimulated an antiviral state *in vivo* that protected mice from lethal influenza virus challenge. This report highlights the therapeutic potential of naturally derived RIG-I agonists as potent stimulators of the innate antiviral response, with the capacity to mobilize genes essential for the generation of efficient immunity against multiple infections.

## Results

### 5′pppRNA stimulates an antiviral response in lung epithelial A549 cells

**Figure 1:**
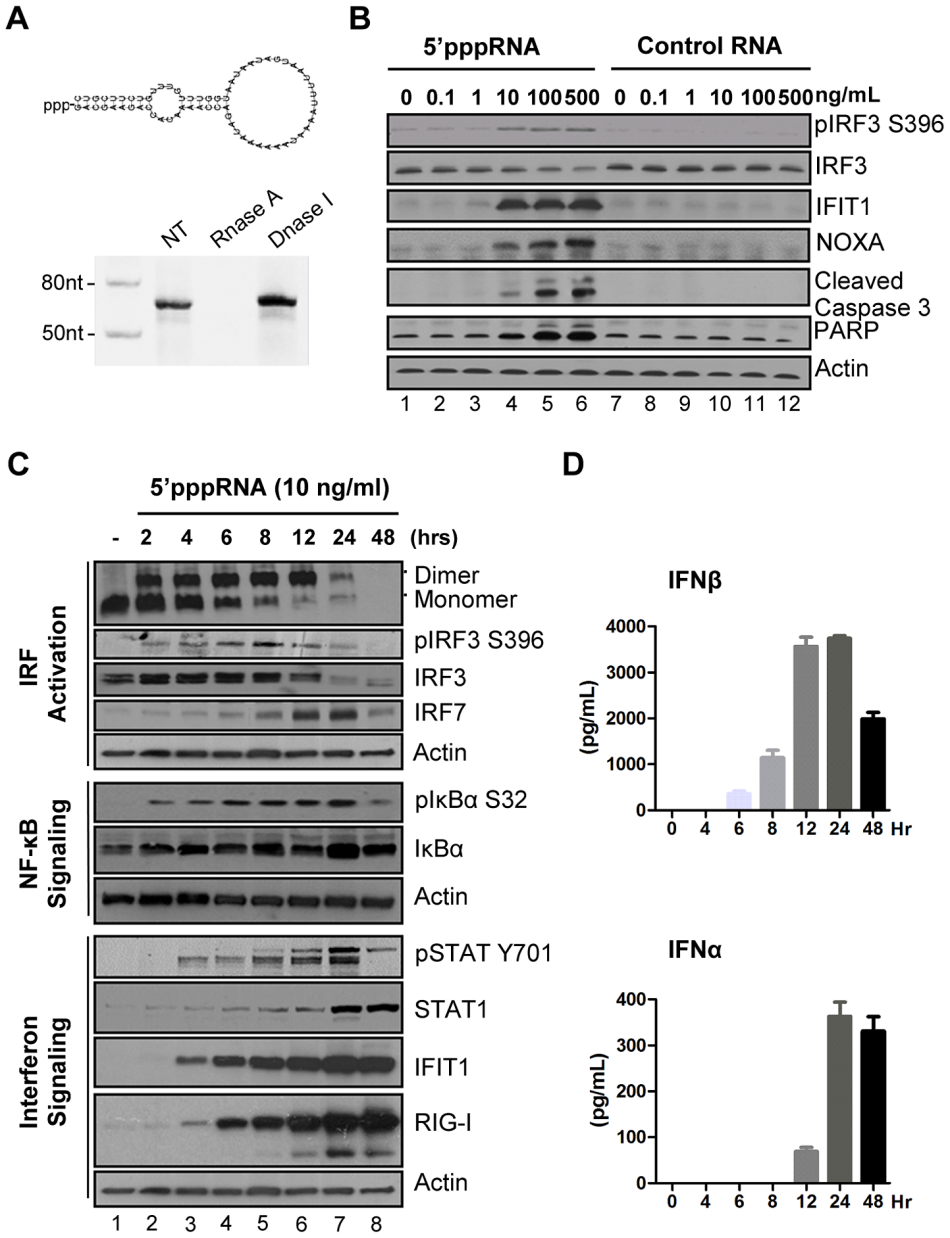
5′pppRNA stimulates an antiviral and inflammatory response in lung epithelial A549 cells. (**A**) Schematic representation of VSV-derived 5′pppRNA and gel analysis. The 5′ppp-containing 67-mer RNA oligonucleotide is derived from the untranslated regions (UTRs) of VSV and the product of *in vitro* transcription runs as a single product degraded by RNase I. (**B**) 5′pppRNA or a homologous control RNA lacking a 5′-triphosphate end was mixed with Lipofectamine RNAiMax and transfected at different RNA concentrations (0.1–500 ng/ml) into A549 cells. At 8 h post treatment, whole cell extracts (WCEs) were prepared, resolved by SDS-page and analyzed by immunoblotting for IRF3 pSer396, IRF3, ISG56, NOXA, cleaved caspase 3, PARP and β-actin. Results are from a representative experiment; all immunoblots are from the same samples. (**C**) A549 cells were transfected with 10 ng/ml 5′pppRNA and WCEs were prepared at different times after transfection (0–48 h), subjected to SDS-PAGE and probed with antibodies for IRF3 pSer-396, IRF3, IRF7, STAT1 pTyr-701, STAT1, ISG56, RIG-I, IκBα pSer-32, IkBα and β-actin; all immunoblots are from the same samples. To detect IRF3 dimerization, WCEs were resolved by native-PAGE and analyzed by immunoblotting for IRF3. (**D**) ELISA was performed on cell culture supernatants to quantify the release of IFNβ and IFNα over time. Error bars represent SEM from two independent samples.

A 5′ triphosphate containing RNA derived from the 5′ and 3′ UTRs of the negative-strand RNA virus Vesicular Stomatitis Virus (VSV) [17] was generated by *in vitro* transcription using T7 polymerase, an enzymatic reaction that synthesizes RNA molecules with a 5′ppp terminus [17]. Predicted panhandle secondary structure of the 5′pppRNA is depicted in Fig. 1A; gel analysis and nuclease sensitivity confirmed the generation of a single RNA product of the expected size (67 nucleotides). Transfection of increasing amounts of 5′pppRNA resulted in Ser396 phosphorylation of IRF3 at 8 h – a hallmark of immediate early activation of the antiviral response (Fig. 1B, lane 2 to 6). Induction of apoptosis was detected following treatment with higher concentrations of 5′pppRNA; the pro-apoptotic BH3-only protein NOXA – a direct transcriptional target of IRF3 [40] – as well as cleavage products of caspase 3 and PARP were up-regulated in a dose dependent manner (Fig. 1B, lane 2–6). Optimal induction of antiviral signaling with limited cytotoxicity was achieved at a concentration of 10 ng/ml (∼500 pM) (Fig. 1B; lane 4). Importantly, the stimulation of immune signaling and apoptosis was dependent on the 5′ppp moiety; a homologous RNA without a 5′ppp terminus abrogated stimulation over a range of RNA concentrations (Fig. 1B, lane 8–12).

To characterize the antiviral response triggered by 5′pppRNA, the kinetics of downstream RIG-I signaling were measured at different times (0–48 h) after stimulation of A549 cells (Fig. 1C). IRF3 homodimerization (1^st^ panel) and IRF3 phosphorylation at Ser396 (2^nd^ panel) were detected as early as 2 h post treatment with 5′pppRNA, and sustained until 24 h. Using a newly characterized anti-IRF7 antibody, induction of endogenous IRF7 was detected with kinetics that was delayed compared to IRF3 activation (4^th^ vs. 3^rd^ panel). IκBα phosphorylation was likewise detected as early as 2 h post-treatment and was sustained in A549 cells (6^th^ panel). Altogether, IRF3, IRF7 and NF-κB are required for optimal induction of the IFNβ promoter [27]. JAK-STAT signaling was detected at 4 h with Tyr701 phosphorylation of STAT1 (9^th^ panel), as well as later accumulation at 24 h (10^th^ panel). IFIT1 and RIG-I itself, were up-regulated 4 h post-treatment (11^th^ and 12^th^ panel) whereas a second group of ISGs (STAT1 and IRF7; 4^th^ and 10^th^ panel) was induced between 6 h and 8 h after agonist treatment. IFNβ was detectable in cell culture supernatant as early as 6 h after 5′pppRNA treatment with a substantial release (4000 pg/ml) that peaked at 12–24 h (Fig. 1D, top panel). IFNα release was detected later at 12 h, and remained high thereafter (400 pg/ml) (Figure 1D). Thus 5′pppRNA triggers a full antiviral response as demonstrated by the activation of transcription factors IRF3, IRF7 and NF-κB, release of interferons, JAK/STAT pathway activation and induction of ISGs.

### 5′pppRNA induction of the antiviral response requires an intact RIG-I pathway

**Figure 2:**
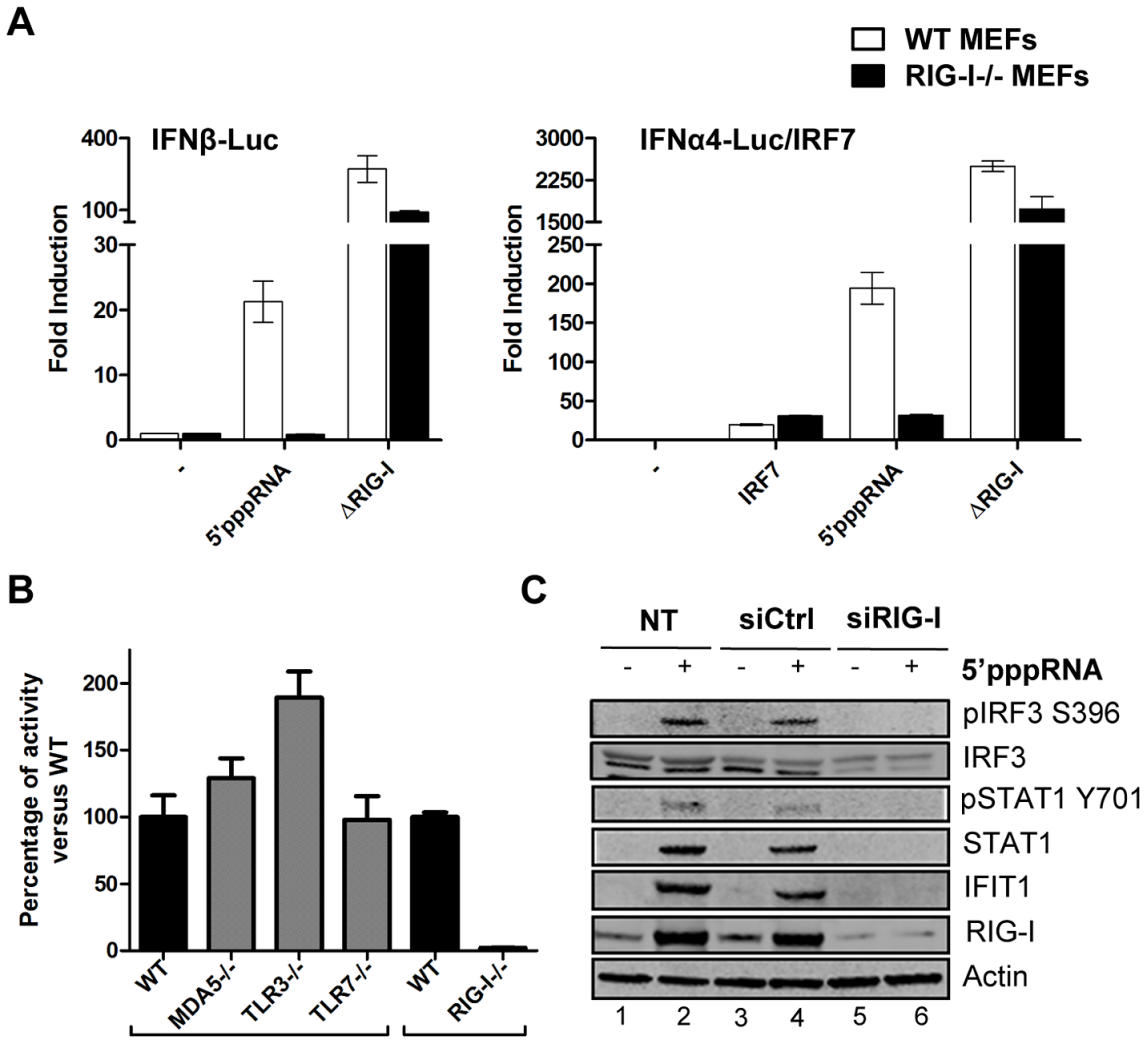
Induction of the interferon response by 5′pppRNA is dependent on functional RIG-I signaling. (**A**) WT and RIG-I−/− MEFs were co-transfected with IFNα4 or IFNβ promoter reporter plasmid (200 ng) along with 5′pppRNA (500 ng/ml) or expression plasmids encoding a constitutively active form of RIG-I (ΔRIG-I) (100 ng). IRF-7 expression plasmid (100 ng) was added for transactivation of the IFNα4 promoter. Luciferase activity was analyzed 24 h post-transfection by the Dual-Luciferase Reporter assay. Relative luciferase activity was measured as fold induction relative to the basal level of reporter gene. Error bars represent SEM from nine replicates performed in three independent experiments. (**B**) Mda5−/−, TLR3−/−, TLR7−/− and RIG-I−/− MEFs were co-transfected with IFNβ promoter reporter plasmid (200 ng) along with 5′pppRNA (500 ng/ml). Luciferase activity was analyzed 24 h post-transfection by the Dual-Luciferase Reporter assay. Relative luciferase activity was measured as fold induction relative to the basal level of reporter gene. Promoter activity in the knockout MEFs was then normalized against the activity in their respective wt MEFs to obtain the percentage of fold activation. Error bars represent SEM from nine replicates performed in three independent experiments. (**C**) A549 cells were either left untreated or transfected with a control siRNA or RIG-I siRNA. After 48 h, 5′pppRNA (10 ng/mL) was transfected and at 8 h after treatment, WCEs were analyzed by SDS-PAGE and immunoblotted for pIRF3 Ser-396, IRF3, pSTAT1 Tyr 701, STAT1, IFIT1, RIG-I, and β-Actin. Results are from a representative experiment; all immunoblots are from the same samples.

To address whether 5′pppRNA exclusively activated the RIG-I sensor, wild type mouse embryonic fibroblasts (wt MEF) and RIG-I−/− MEF were co-transfected with 5′pppRNA and type 1 IFN reporter constructs to measure promoter activity. 5′pppRNA activated the IFNβ promoter 60-fold and the IFNα promoter 450-fold in wt MEF; promoter activity was dependent on RIG-I since these promoters were not stimulated in RIG-I−/− MEF. As positive control, a constitutively active, CARD domain-containing RIG-I mutant [41] was used to bypass the requirement for RIG-I (Figure 2A). Furthermore, induction of the IFN response was exclusively dependent on intact RIG-I signaling, since IFNβ promoter activity was not decreased in Mda5−/−, TLR3−/− or TLR7−/− MEFs (Figure 2B). In A549 cells treated with 5′pppRNA, knocking down RIG-I abolished IRF3 and STAT1 phosphorylation, as well as IFIT1 and RIG-I upregulation compared to control siRNA-treated cells (Fig. 2C; lane 6 vs. 4). Of note, generation of the knock down by transient transfection of short RNA did not activate immune signaling (Fig. 2C; lane 3 vs. 1). Hence, this 5′pppRNA signals specifically via RIG-I.

### Kinetics of the host response to 5′pppRNA

**Figure 3:**
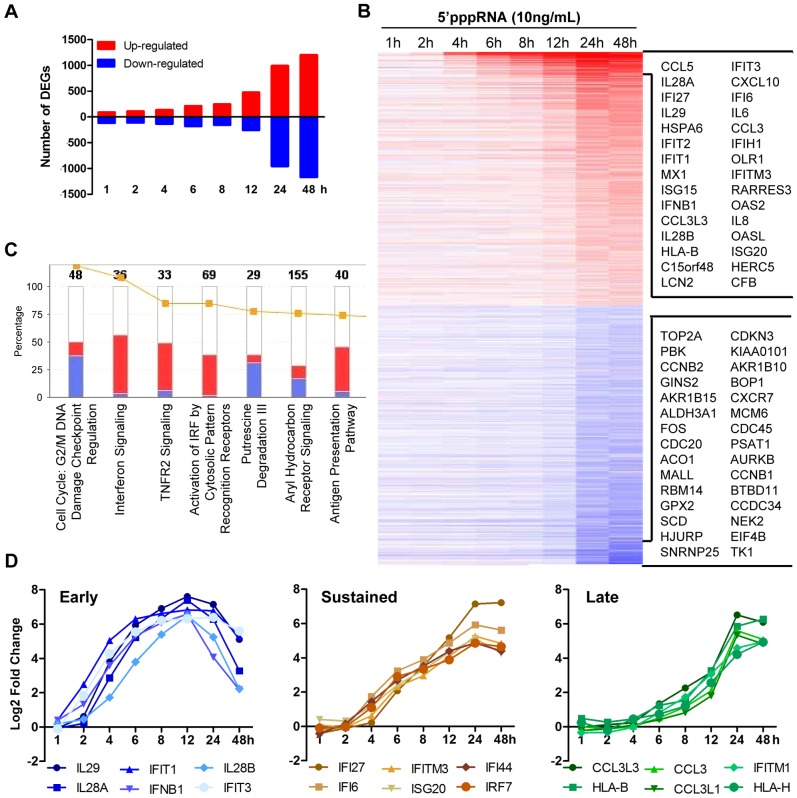
Transcriptome analysis of the host antiviral response to 5′pppRNA. A549 cells were transfected with 10 ng/ml of 5′pppRNA using Lipofectamine RNAiMax for designated periods of time. Samples were analyzed by Illumina gene expression array and DEG were identified based on fold change ≥±2 and *p*-value ≤0.001 (**A**) Number of up-regulated and down-regulated DEG at each time point. (**B**) Heatmap of all DEG sorted by fold change; top 30 genes are listed. Red, Up-regulated; blue, down-regulated. (**C**) Functional characterization of DEGs following 5′pppRNA treatment based on Ingenuity Pathway Analysis software. Bar height refers to the number of DEG in each pathway and the color refers to the contribution from up-regulated or down-regulated genes. (**D**) Genes among the top up-regulated genes were selected based on three different expression patterns: early, sustained, late.

To evaluate the breadth of the host intrinsic response resulting from RNA agonist stimulation of RIG-I, modulation of the transcriptome of A549 cells stimulated with 5′pppRNA from 1 to 48 h was analysed by gene array using the Illumina platform. Figure 3A shows a waterfall plot of differentially expressed genes (DEG; selected based on fold change ≥±2, *p*-value ≤0.001) after 5′pppRNA stimulation. The number of genes up-regulated by 5′pppRNA administration steadily increased with time, while the majority of down-regulation occurred at 24–48 h (Fig. 3A). The heatmap presents DEG with emphasis on the most highly deregulated genes over time (Fig. 3B). Canonical pathway analysis using Ingenuity Pathway Analysis software identified IFN signaling, activation of IRFs by cytosolic PRRs, TNFR2 signaling and antigen presentation as the main up-regulated functional categories, while functions related to cell metabolism and cell cycle were down-regulated by RIG-I agonist treatment (Fig. 3C). Subsequent kinetic analysis revealed that RIG-I agonist induced distinct temporal patterns of gene expression (Fig. 3D, S1 and S2A). For example, some genes were highly expressed early at 6–12 h, including *IFNB1* and the IFNλ family (*IL29*, *IL-28A*, *IL28B*), but the expression of these genes was not sustained throughout the time course and decreased at 24–48 h (Fig. 3D; lest panel). A second subset of genes associated with the antiviral response were induced early at 6–8 h, but expression was sustained and markedly augmented at 24–48 h, as exemplified by *IFI* family members, *IRF7*, and other ISGs (Fig. 3D; middle panel). A third subset of genes was induced primarily at later time points, as part of the secondary response to 5′pppRNA treatment, and included *HLA* and *CCL3* families (Fig. 3D; right panel). Representative genes from different subsets were validated by quantitative real-time RT-PCR (Fig. S1). Overall, 5′pppRNA induced a robust bi-phasic transcriptional response, characterized by strong activation of antiviral and inflammatory gene signatures; the kinetics of the transcriptional profile mirrored the biochemical activation events detected in Figures 1.

**Figure 4:**
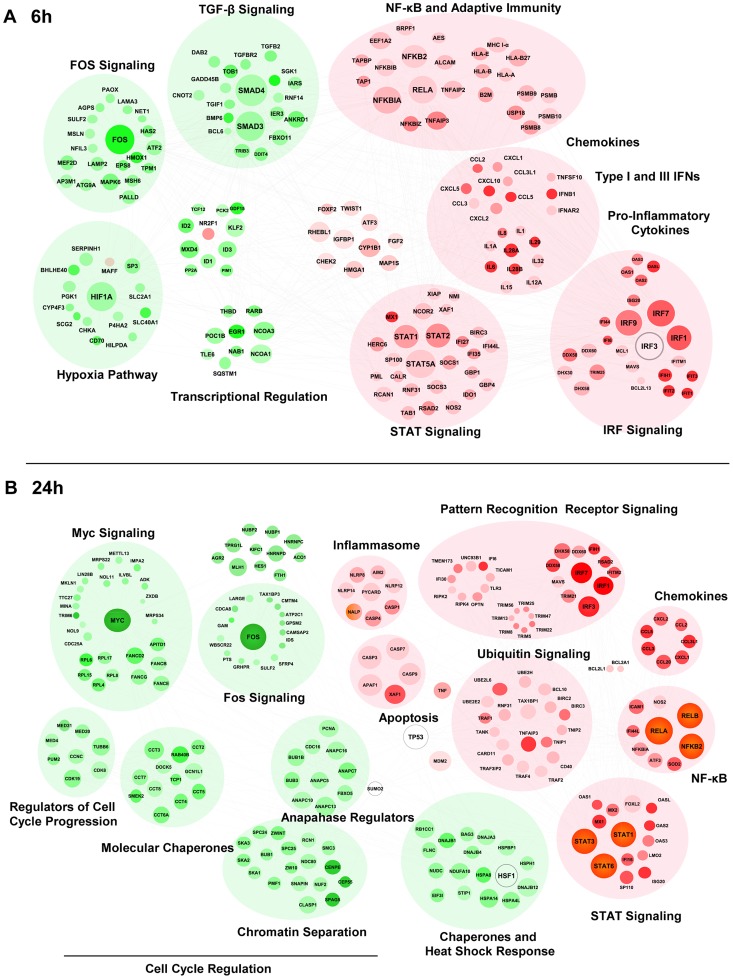
Functional characterization of genes differentially expressed by RIG-I. The 6 h (**A**) and 24 h (**B**) time points from the kinetic analysis were selected to perform a functional classification of the up-regulated (red) and down-regulated (green) genes by 5′pppRNA. Genes with a fold change ≥±2 and *p*-value ≤0.001 were clustered using IPA. The intensity of the color is representative of the fold change. Larger circles indicate a transcription factor.

In order to gain systems-wide insight into the RIG-I transcriptome, a functional clustering of 5′pppRNA-induced DEGs was performed. This functional clustering identified a variety of transcriptional sub-networks and biological processes regulated by RIG-I (Fig. 4). As expected, at 6 h (Fig. 4A), induction of antiviral and inflammatory response programs downstream of IRF, NF-κB, STAT signaling were identified (Fig. 4A and S2B); expression of several cytokine and chemokine genes were also up-regulated. Concomitantly, genes related to Fos and TGF-β signaling, as well as hypoxic signaling via HIF-1α were down-regulated. At 24 h (Fig. 4B), genes associated with pathogen recognition receptor signaling, the ubiquitin pathway, inflammation and apoptosis were also induced by RIG-I activation; interestingly, profiling of down-regulated genes identified functional clusters involved in cell cycle regulation, MYC signaling, and the heat shock response.

### Comparative analysis of the gene expression profile in response to 5′pppRNA and IFNα-2b

**Figure 5:**
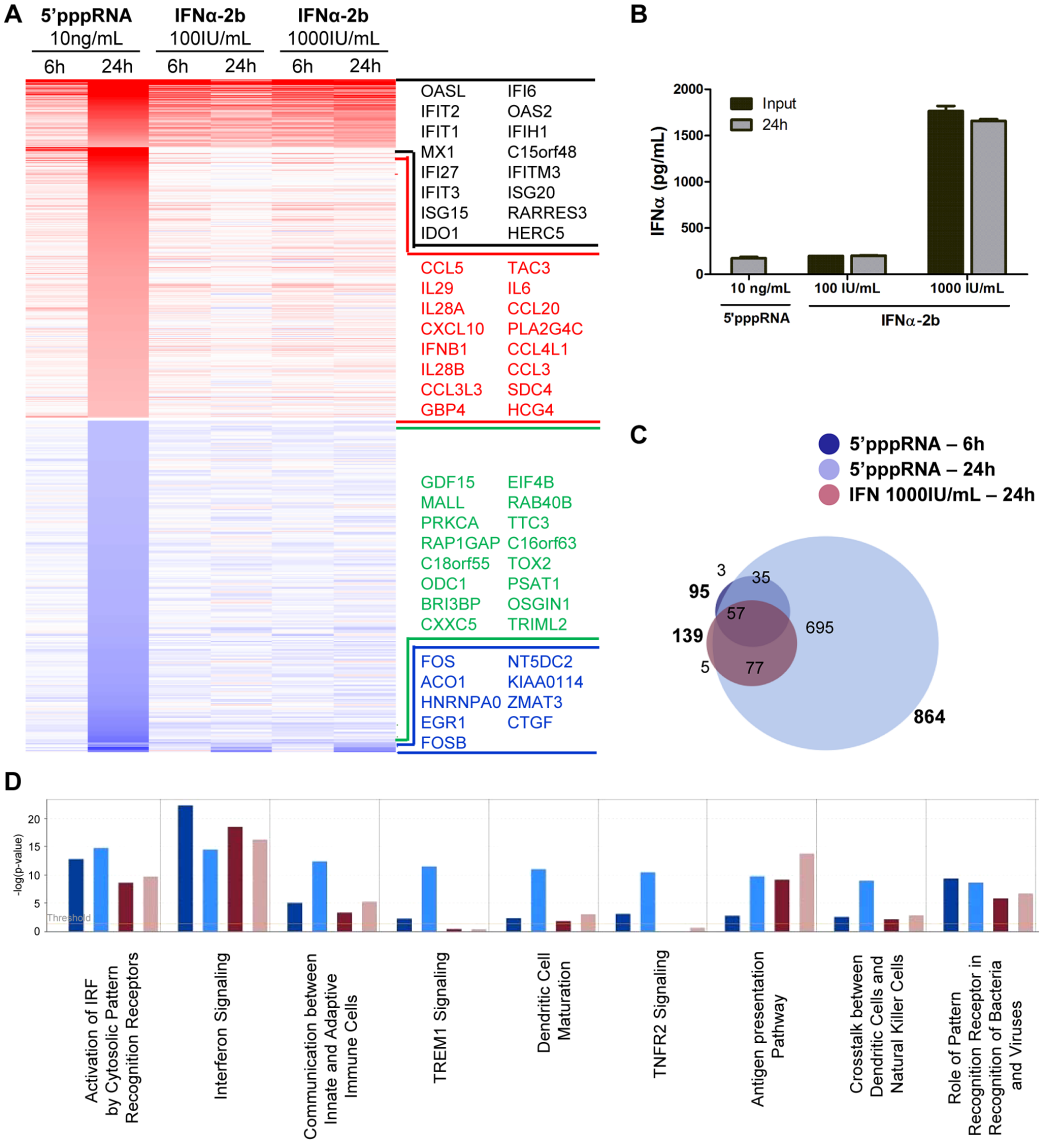
Gene expression profiling of differentially expressed genes in response to 5′pppRNA and IFNα-2b. A549 cells were transfected with 10 ng/ml of 5′pppRNA using Lipofectamine RNAiMax or treated with IFNα-2b (100 IU/ml or 1000 IU/ml). Samples were collected at 6 h or 24 h post-treatment and were analyzed by Illumina gene expression array. Genes with a fold change ≥±2.0 and *p*-value ≤0.001 were considered differentially expressed. (**A**) Heatmap showing top DEG affected by 5′pppRNA and IFNα-2b treatments. Genes regulated by both 5′pppRNA and IFNα-2b in at least one condition are indicated in black (up-regulated) or blue (down-regulated). Genes uniquely induced by 5′pppRNA in at least one time point but not by IFNα-2b in any conditions are highlighted in red (up-regulated) or green (down-regulated). Top genes are listed in each instance. (**B**) Cell culture supernatant was collected at the time of treatment with IFNα-2b (0 h; input), or at 24 h following 5′pppRNA or IFNα-2b treatment and assayed by ELISA for multiple subunits of IFNα. Error bars represent SEM from two independent samples. (**C**) Surface proportional Venn diagram illustrating the magnitude of the response by 5′pppRNA and IFNα-2b at 6 h and 24 h. Number of DEG is indicated in each area. (**D**) Comparison of genes induced by each treatment - 5′pppRNA 6 h (dark blue); 5′pppRNA 24 h (light blue); IFNα-2b (1000 IU/ml) 6 h (red); IFNα-2b (1000 IU/ml) 24 h (pink) - based on functional classification by Ingenuity Pathway Analysis.

Although the contribution of type I IFN to the antiviral response stimulated by 5′pppRNA is unquestionable, other factors may also augment the antiviral state established by 5′pppRNA. To define gene uniquely induced by 5′pppRNA, gene expression profiles of A549 cells stimulated with 5′pppRNA or with IFNα-2b for 6 and 24 h were compared. The heatmap in Figure 5A displays the expression profile of genes differentially up- and down-regulated (fold change ≥2; *p*-value ≤0.001) by 5′pppRNA and IFN (black and blue genes) or exclusively by 5′pppRNA (red and green genes). Interestingly, 5′pppRNA induced a significantly broader gene expression program compared to IFNα-2b, especially at 24 h. To determine whether differences in gene expression observed between 5′pppRNA and IFNα-2b were due to sub-optimal stimulation by IFNα-2b, higher concentrations of IFNα-2b within the range reported for *in vitro* applications were tested. Treatment with 5′pppRNA at 10 ng/ml was equivalent to treatment with IFNα-2b at 100 IU/ml in terms of IFNα levels released into cell culture supernatant. Increasing the concentration of IFNα-2b to 1000 IU/ml corresponded to levels of IFNα that were 8-times greater than physiological secretion following 10 ng/ml 5′pppRNA treatment (2500 pg/ml vs 480 pg/ml; Fig. 5B). Regardless of the amount of IFNα-2b used to activate cells, gene expression levels remained relatively unchanged (Fig. 5A; IFNα-2b 100 IU/ml vs 1000 IU/ml), indicating that IFNα-2b treatment was saturating.

Remarkably, the spectrum of genes differentially expressed by IFNα-2b treatment were virtually all contained within the transcriptome induced by 5′pppRNA treatment - 57 at 6 h and 134 at 24 h, out of 139 genes (Fig. 5C) - demonstrating that 5′pppRNA induced a complete IFN response by 24 h. Some of the genes characteristically activated as part of the IFN signature and maximally induced by 5′pppRNA are *MX1*, *IFIT1*, *ISG15* (Fig. 5A, black portion). This comparison also highlighted the fact that a surprisingly large number of genes are uniquely regulated by 5′pppRNA - 38 genes at 6 h and 730 genes at 24 h (Fig. 5C). Most notably, *IFNB1* and all three members of the IFNλ family, as well as the cytokines *CCL5*, *CXCL10*, *IL-6*, and *CCL3*, were highly induced by 5′pppRNA but not IFNα-2b treatment (Fig. 5A; red genes). While IFN signaling was highly induced by both treatments, IFNα-2b strongly activated antigen presentation machinery, and 5′pppRNA preferentially stimulated dendritic cell maturation and crosstalk, linking innate and adaptive immunity as well as induction of a wider range of signaling pathways (Fig. 5D). Significantly, 5′pppRNA preferentially stimulated a more extensive induction of IRF7 and NF-κB signaling nodes compared to IFNα-2b treatment (Fig. S2C). Thus, 5′pppRNA treatment, besides inducing a complete IFN response, additionally stimulated the transcription of a large and unique set of inflammatory and antiviral genes.

### 5′pppRNA acts as a broad-spectrum antiviral agent

**Figure 6:**
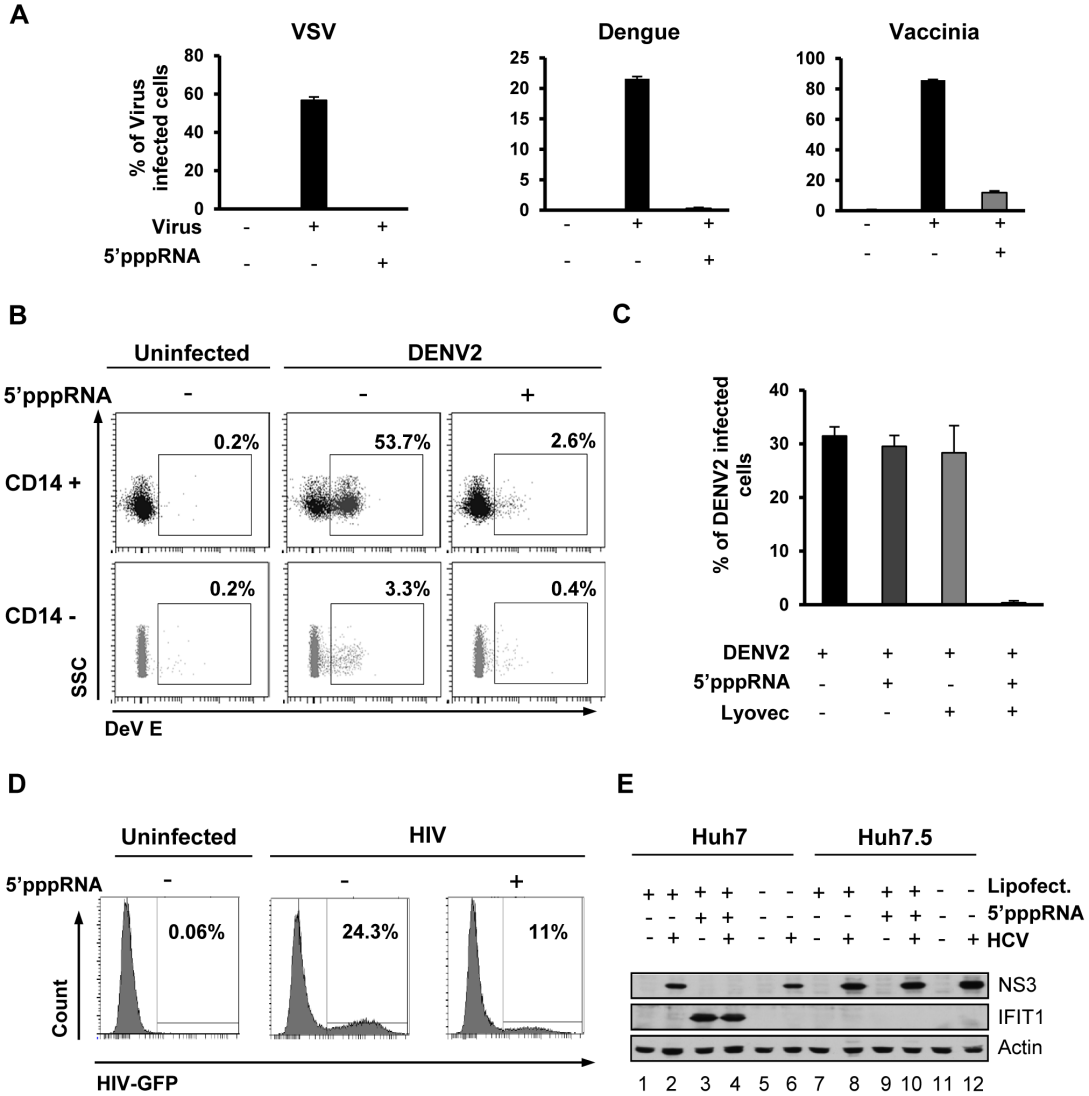
5′pppRNA acts as a broad-spectrum antiviral agent. (**A**) A549 cells were transfected with 10 ng/ml 5′pppRNA 24 h prior to infection with VSVΔ51-GFP (MOI 0.1), Dengue virus (MOI 0.1), and Vaccinia-GFP virus (MOI 5), respectively. Percentage of infected cells was determined 24 h post-infection by flow cytometry analysis of GFP expression (VSV-GFP and Vaccinia-GFP) or intracellular staining of DENV E protein expression (Dengue virus). Data are from a representative experiment performed in triplicate ± SD. (**B**) Human PBMCs were transfected with 100 ng/ml 5′pppRNA 24 h prior to infection with dengue virus at an MOI of 5. At 24 h post-infection, the percentage of Dengue infected CD14+ and CD14− cells was evaluated by intracellular staining of DENV E protein expression by flow cytometry. Data are from a representative experiment performed in triplicate ± SD. (**C**) Human PBMCs from three different donors were transfected with 100 ng/ml 5′pppRNA prior to infection with Dengue virus at an MOI of 5. The percentage of Dengue infected cells in the CD14+ population was evaluated by intracellular staining of DENV E protein expression using flow cytometry. Data are from an experiment performed in triplicate on three different patients ± SD (**D**) CD4+ T cells isolated from human PBMCs and activated with anti-CD3 and anti-CD28 antibodies. Cells were incubated in the presence or absence of supernatant from 5′pppRNA-treated monocytes for 4 h and infected with HIV-GFP (MOI 0.1) for 48 h. The percentage of HIV infected, activated CD4+ T cells (GFP positive) was assessed by flow cytometry. (**E**) Huh7 and Huh7.5 were transfected with 5′pppRNA (10 ng/mL) and infected with HCV 24 h later. At 48 h post-infection, WCEs were collected and subjected to SDS PAGE and immunoblot to examine the expression of HCV viral protein NS3, IFIT1, and β-Actin.

To determine if the RIG-I agonist was capable of inducing a functional antiviral response, A549 cells were treated with 5′pppRNA, and 24 h later, challenged with VSV, Dengue (DENV), or Vaccinia viruses. All viruses established infection in untreated cells as assessed by flow cytometry (60%, 20% and 80%, respectively) but in 5′pppRNA-treated cells, VSV and DENV infectivity was reduced to <0.5%, while infection with vaccinia was reduced to 10% (Fig. 6A). Release of infectious VSV and DENV virus was completely blocked by 5′pppRNA treatment (1.7×10^9^ and 4.3×10^6^ PFU/mL in untreated cells, respectively vs. undetectable in treated cells; Fig. S3). Similarly, in primary human CD14+ monocytes, DENV infection decreased from 53.7% to 2.6% in the presence of 5′pppRNA; in the CD14− fraction, DENV infectivity was lower (3%), but was likewise inhibited by RNA agonist treatment (0.4%; Fig. 6B). To demonstrate the requirement for intracellular delivery of 5′pppRNA, primary CD14+ cells from three patients were treated with 5′pppRNA alone, transfection reagent alone or the combination; DENV infectivity was reduced from ∼30% to ∼0.5% only upon transfection of the RNA agonist (Fig. 6C). To evaluate the antiviral effect of 5′pppRNA against HIV infection, activated CD4+ T cells were pre-treated with supernatant isolated from 5′pppRNA-treated monocytes and then infected with HIV-GFP. In the absence of treatment, 24% of the activated CD4+ T cells were actively infected by HIV as determined by GFP expression by flow cytometry, whereas infection of CD4+ T cells treated with 5′pppRNA supernatants was reduced to 11% (Fig. 6D). 5′pppRNA also had an antiviral effect against HCV in hepatocellular carcinoma cell line Huh7; HCV NS3 expression was inhibited by 5′pppRNA treatment (Fig. 6E; lane 4 vs. 2 and 6). The antiviral effect was fully dependent on RIG-I, as demonstrated in Huh7.5 cells (that express a mutated inactive RIG-I) by the absence of IFIT1 up-regulation following 5′pppRNA treatment (Fig. 6E; lane 9) and NS3 expression comparable to untreated HCV-infected cells (Fig. 6E; lane 10 vs. 8 and 12). Thus, 5′pppRNA is a broad-spectrum antiviral agent able to trigger an efficient innate immune response in different cell types and prevent infection by RNA and DNA viruses.

### 5′pppRNA inhibits H1N1 Influenza infection *in vitro*

**Figure 7:**
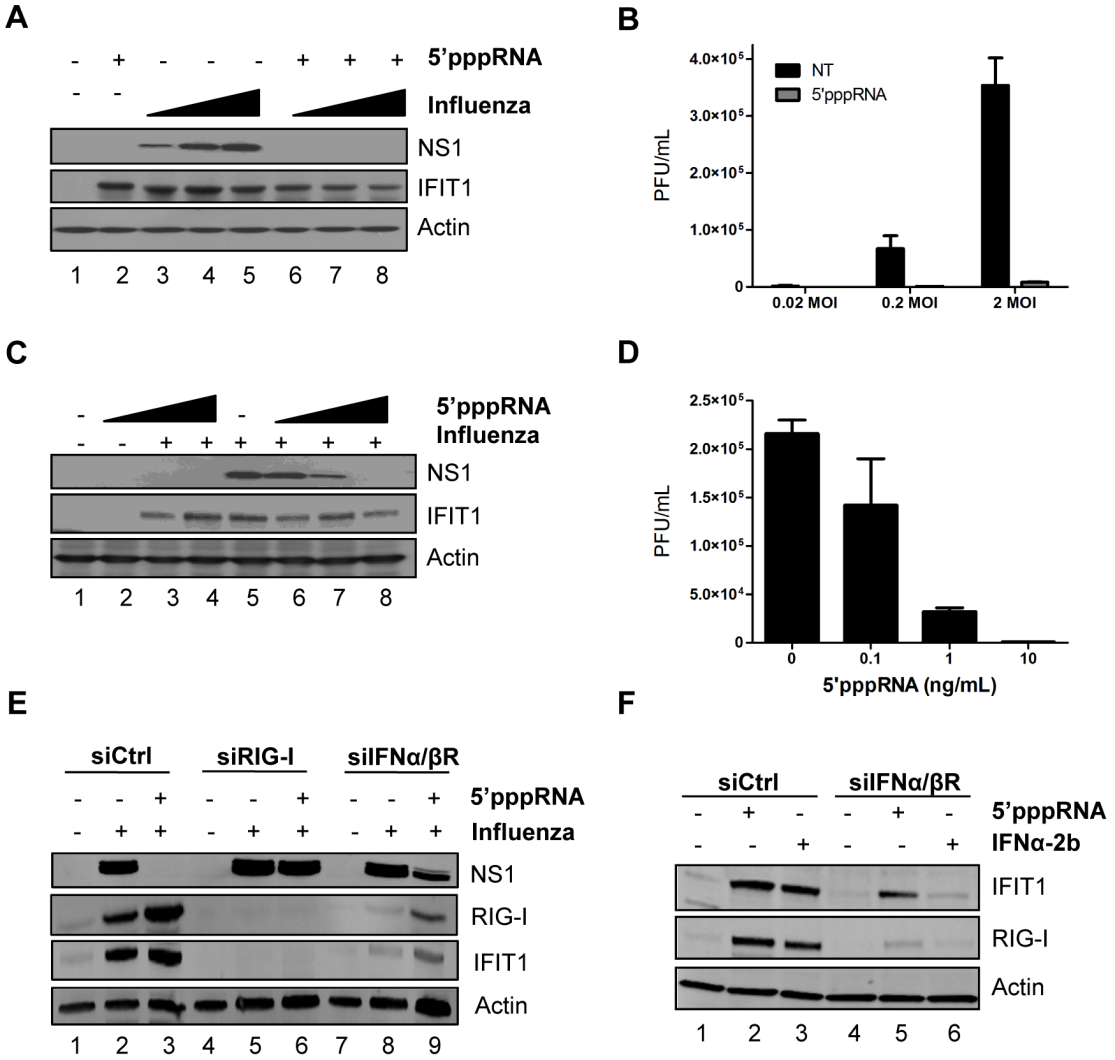
5′pppRNA inhibits H1N1 Influenza replication *in vitro*. (**A**) A549 cells were treated with 5′pppRNA (10 ng/ml); at 24 h post-treatment, cells were infected with an increasing MOI of A/PR8/34 H1N1 Influenza virus (0.02MOI, 0.2MOI, 2MOI) for 24 h. WCEs were subjected to SDS PAGE and immunoblot to examine the expression of influenza viral protein NS1, ISG56, and β-Actin. (**B**) Viral titers in cell culture supernatants from (A) were determined by plaque assay. Error bars represent SEM from two independent samples. (**C**) A549 cells were pre-treated with increasing concentrations of 5′pppRNA (0.1 ng/ml to 10 ng/ml) for 24 h, prior to 0.2MOI influenza challenge. WCEs were examined for influenza NS1, ISG56, β-Actin by immunoblot. (**D**) Viral titers in cell culture supernatants from (C) were determined by plaque assay. Error bars represent SEM from two independent samples. (**E**) A549 cells were transfected with a control siRNA, RIG-I siRNA or IFNα/βR siRNA and then treated with 5′pppRNA (10 ng/ml), followed by infection with Influenza (0.2MOI). WCEs were prepared 24 h later and subjected to SDS PAGE and immunoblot to examine the expression of influenza NS1, RIG-I, IFIT1, and β-Actin. (**F**) A549 cells were transfected with a control siRNA or IFNα/βR siRNA and then treated with 5′pppRNA (10 ng/ml) or IFNα-2b (100 IU/mL) for 24 h. Expression of IFIT1, RIG-I and β-Actin was evaluated by western blotting.

To further explore the inhibitory potential of 5′pppRNA, A549 cells were pre-treated with 5′pppRNA for 24 h and then infected with H1N1 A/PR/8/34 Influenza virus at increasing MOI (0.02, 0.2, 2); influenza replication was monitored by NS1 protein expression (Fig. 7A) and plaque assay (Fig. 7B). Viral replication was blocked by 5′pppRNA pre-treatment, even at the highest MOI, as demonstrated by complete loss of NS1 expression and 40-fold decrease in viral titer at MOI 2. In A549 cells pre-treated with decreasing concentrations of 5′pppRNA (10–0.1 ng/ml) prior to influenza virus challenge (0.2 MOI), 5′pppRNA significantly blocked influenza replication at a concentration of 1 ng/ml, as demonstrated by a 3-fold reduction in NS1 protein expression (Fig. 7C; lane 7) and a 7-fold reduction in virus titer by plaque assay (Fig. 7D).

To demonstrate that the antiviral activity of 5′pppRNA against influenza relies on RIG-I signaling, A549 cells were knocked down for RIG-I and infected with influenza; in the knockdown, ISGs were not induced (Fig. 7E, lanes 3 vs. 6) and 5′pppRNA treatment failed to inhibit NS1 expression (Fig. 7E; lanes 5 vs. 6), indicating that the antiviral effect of 5′pppRNA is exclusively dependent on RIG-I. Next, to determine whether the RIG-I ‘unique’ gene expression profile characterized in Figure 5 could compensate for the IFN response, A549 cells were knocked down for the IFNα/βR; the knock down was efficient, as demonstrated by the absence of IFIT1 and RIG-I induction following IFNα-2b stimulation (Fig. 7F; lane 6). Interestingly, induction of ISGs was only partially reduced following 5′pppRNA treatment (2.2-fold reduction of IFIT1 vs. siCtrl; Fig. 7F; lane 5 vs. 2); this IFN-independent activation of innate signaling was sufficient to reduce viral NS1 expression by 2.4-fold (Fig. 7E; lane 9 vs. 8). Thus, in lung epithelial A549 cells, 5′pppRNA treatment can efficiently inhibit influenza H1N1 replication in a RIG-I-dependent manner and stimulate an antiviral and inflammatory response independently of IFN signaling to limit influenza infection *in vitro*.

### 5′pppRNA activates innate immunity and protects mice from lethal influenza infection

To determine the potential of 5′pppRNA *in vivo*, C57Bl/6 mice were inoculated intravenously with 5′pppRNA (25 µg) in complex with the *in vivo*-jetPEI transfection reagent. 5′pppRNA stimulated a potent immune response *in vivo* characterized by IFNα and IFNβ secretion in the serum and lungs (Fig. S4A) as well as antiviral gene up-regulation (Fig. S4B). The response was potent and rapid with serum IFNβ levels increased ∼20-fold compared to basal levels, as early as 6 h post administration (Fig. S4A; top left panel). The immune activation observed *in vivo* correlated with an early and transient recruitment of neutrophils to the lungs along with a more sustained increase in macrophages and dendritic cells populations (Fig. S4C).

**Figure 8:**
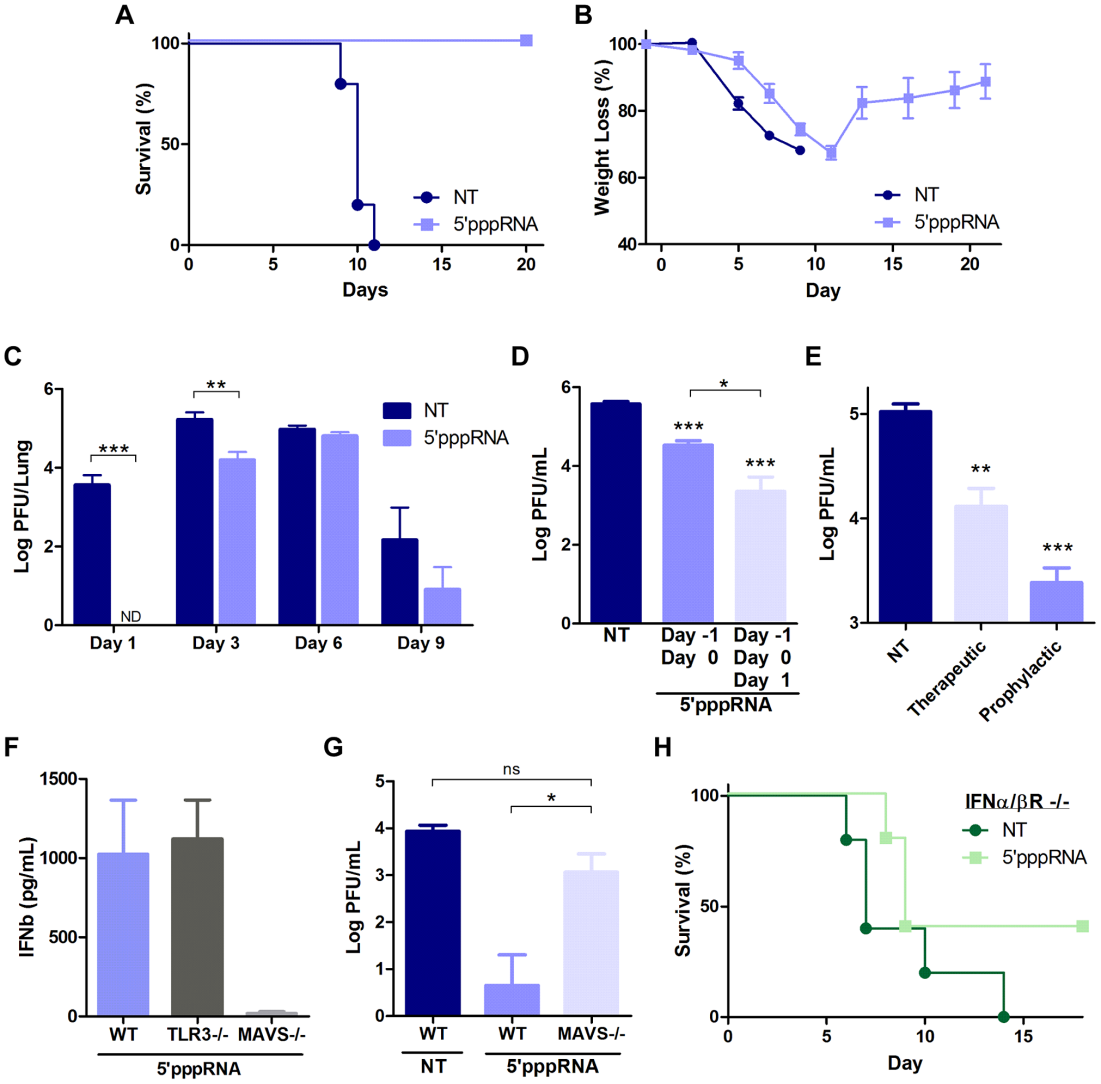
5′pppRNA activates innate immunity and protects mice from lethal influenza infection *in vivo*. C57Bl/6 mice were injected intravenously with 25 ug of 5′pppRNA in complex with in vivo-jetPEI. (**A–B–C**) Mice were treated with 5′pppRNA on the day prior to influenza infection (500 PFU; Day −1) and the day of infection (Day 0). Percent survival (**A**) and percent weight loss (**B**) were monitored. (**C**) Lung viral titers were measured by plaque assay at the indicated day post-infection. Error bars represent SEM from six different animals. ND: not detected. (**D**) Repetitive administration of 5′pppRNA on the indicated days further decreases lung viral titers following infection with 500 PFU of influenza, as determined by plaque assay on Day 3 post-infection. Error bars represent SEM from five different animals. (**E**) Mice were infected with 50 PFU of influenza on Day 0. 5′pppRNA was administered prophylactically (Day −1, Day 0) or therapeutically (Day 1, Day 2) and lung viral titers were determined on Day 3. Error bars represent SEM from five different animals. (**F**) WT, TLR3−/−, MAVS−/− mice were treated with 5′pppRNA and serum IFNβ was quantified by ELISA at 6 h. Error bars represent SEM from three different animals. (**G**) WT and MAVS−/− mice were treated with 5′pppRNA and infected with influenza (500 PFU). Lungs were collected and homogenized on Day 1 and lung viral titers were determined by plaque assay. Error bars represent SEM from four different animals. (**H**) IFNα/βR−/− mice were either non-treated or treated with 5′pppRNA and infected with influenza (100 PFU). Survival was monitored for 18 days. Statistical analysis was performed by Student's t test (*, p≤0.05; **, p≤0.01; ***, p≤0.001; ns, not statistically significant).

Next, to determine the antiviral potential of 5′pppRNA *in vivo*, mice were treated with 5′pppRNA 24 h before (day −1), and on the day of infection (day 0) with a lethal inoculum of H1N1 A/PR/8/34 Influenza. Whereas all untreated, infected mice succumbed to infection by day 11, all 5′pppRNA-treated mice fully recovered (100% survival) (Fig. 8A). Overall, weight loss was similar between the two groups (Fig. 8B), although a noticeable delay of 2–3 days in the onset of weight loss was observed in 5′pppRNA-treated animals; treated mice then fully recovered within 12–14 days (Fig. 8B). Influenza replication in the lungs was monitored by plaque assay over the course of infection with virus titers in the lungs of untreated mice reaching a maximum at day 3 post-infection (Fig. 8C). A decrease in virus titer was noted by day 9 post-infection, possibly correlating with the onset of adaptive immunity, and all animals succumbed to influenza infection by day 11 (Fig. 8A). Interestingly, 5′pppRNA treatment inhibited influenza virus replication in the lungs early after infection, within the first 24–48 h (Fig. 8C; Day 1); by day 3, virus titers in the lung had increased, although influenza titers were still ∼10-fold lower compared to titers in untreated mice (Fig. 8C; Day 3). By day 9, the 5′pppRNA-treated animals had controlled the infection, as demonstrated by the decrease in viral titers. Continuous administration of 5′pppRNA at 24 h intervals post-infection had an additive therapeutic effect that further delayed viral replication (Fig. 8D; 3 versus 2 doses of 5′pppRNA), indicating that antiviral immunity may be sustained with repetitive administration of 5′pppRNA. Furthermore, therapeutic administration of 5′pppRNA also controlled influenza viral replication; although prophylactic treatment was most effective at blocking influenza dissemination in the lung, administration of the RNA agonist on day 1 and day 2 after influenza infection also reduced viral lung titers by ∼10-fold (Fig. 8E).

The antiviral response triggered by 5′pppRNA *in vivo* was dependent on an intact RIG-I signaling; serum IFNβ release was abolished in MAVS−/− mice, whereas the absence of TLR3 did not affect 5′pppRNA-induced IFNβ release (Fig. 8F). In agreement, MAVS−/− mice treated with 5′pppRNA did not control influenza lung titers (5-fold increase vs. wt mice) and the titer was comparable to untreated wt mice (Fig. 8G). To determine whether 5′pppRNA treatment was sufficient to protect against influenza in the absence of IFN signaling, IFNα/βR−/− mice were treated or not with 5′pppRNA and challenged with influenza H1N1 virus. While untreated IFNα/βR−/− animals succumbed to infection, 40% of the animals that received 5′pppRNA treatment survived, suggesting that an IFN-independent effect of 5′pppRNA functioned in the absence of the IFN response. Thus, intravenous administration of 5′pppRNA stimulated a potent and rapid immune response *in vivo* that delayed influenza H1N1 virus replication in the lungs of infected animals and rescued mice from a lethal inoculation of influenza H1N1.

### 5′pppRNA treatment limits influenza-mediated pneumonia

**Figure 9:**
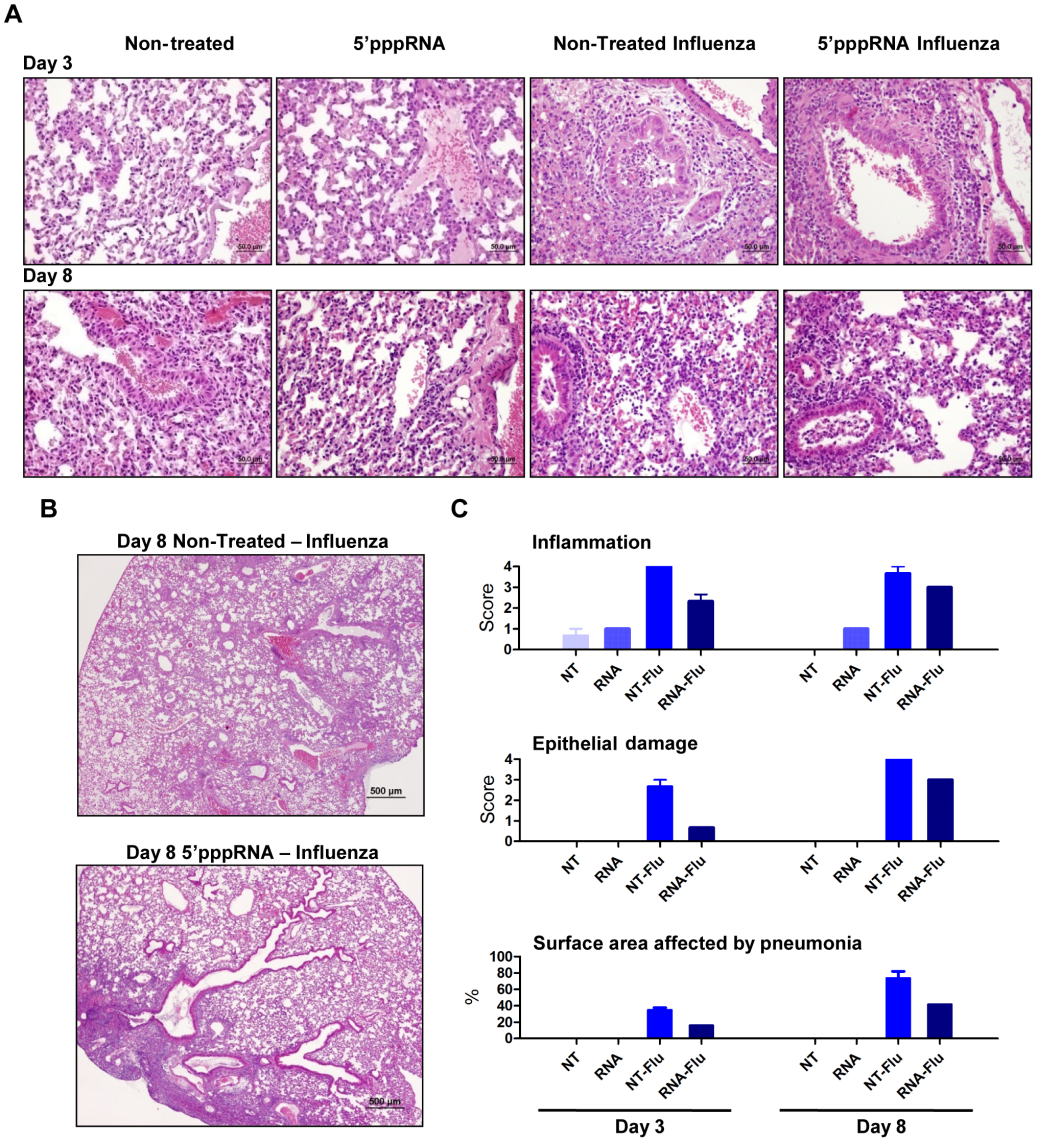
5′pppRNA treatment controls influenza-mediated pneumonia. Mice were treated with 5′pppRNA in complex in vivo-jetPEI on the day prior to influenza infection (Day −1) and the day of infection (Day 0). Lungs were collected on Day 3 and Day 8 post-infection and stained with hematoxylin and eosin (H&E). For each group, a representative picture showing (**A**) inflammation and tissue damage and (**B**) the extent of pneumonia is presented. (**C**) Inflammation, tissue damage and surface area affected by pneumonia were scored by a veterinary pathologist. Grade 1 = minimal; Grade 2 = modest, rare; Grade 3 = moderate, frequent; Grade 4 = severe, extensive.

To further evaluate the effect of RNA agonist administration on influenza-mediated pathology, histological sections of lungs from untreated and treated mice were prepared and analysed. 5′pppRNA treatment alone was characterized by a modest and rare leukocyte-to-endothelium attachment; mixed leukocyte populations (mononuclear/polymorphonuclear) infiltrated the perivascular space at 24 h after injection (data not shown) but the infiltration resolved and was limited to endothelial cell attachment at 3 and 8 days after intravenous administration (Fig. 9A). Influenza virus infection induced severe and extensive inflammation and oedema in the perivascular space and the bronchial lumen at day 3 post-infection. In animals receiving the RNA agonist, influenza triggered a mild and infrequent inflammation that did not extend to the bronchial lumen at day 3 post-infection. Epithelial degeneration and loss of tissue integrity were more severe in the lungs of untreated, infected animals and correlated with epithelial hyperplasia observed at later times, compared to the lungs of animals treated with 5′pppRNA. Inflammation and epithelial damage progressed in untreated mice by day 8 (Fig. 9E), and correlated with increased virus titer in the lungs (Fig. 8C); inflammation and epithelial damage was consistently less apparent in agonist-treated mice. Strikingly, the surface area of the lungs affected by pneumonia was significantly reduced in 5′pppRNA-treated mice compared to non-treated mice – on day 3, 16% vs 35%; day 8, 41% vs 73% (Fig. 9C; bottom panel). Overall, influenza-mediated pneumonia was less severe in animals administered 5′pppRNA before influenza challenge, demonstrating that 5′pppRNA possesses an antiviral effect *in vivo* that limits influenza replication in the lung, limits lung damage and prevents influenza-mediated pneumonia and mortality.

## Discussion

RIG-I agonists are attractive potential antiviral agents, as triggering the innate cytosolic RIG-I pathway mimics the earliest steps of immune recognition and response to viral pathogens. In the present study, a short 5′pppRNA agonist of RIG-I derived from the 5′ and 3′ UTRs of the VSV genome stimulated an antiviral response that protected human lung epithelial A549 cells or human PBMCs from challenge with several viruses, including DENV, Influenza, HIV, VSV, HCV and Vaccinia virus. Intravenous administration of the 5′pppRNA agonist in mice stimulated an antiviral state *in vivo* that protected animals from lethal influenza virus challenge and controlled influenza virus-mediated pneumonia. Analysis of the dynamics of the host transcriptome following 5′pppRNA stimulation was characterized by antiviral and inflammation related gene expression patterns with transcriptional nodes of genes regulated by IRF, NF-κB, and STAT families. Virtually all of the genes activated by IFNα-2b were encompassed within the 5′pppRNA transcriptome; bioinformatics analysis also identified distinct gene patterns and functional processes that were uniquely induced or inhibited by 5′pppRNA. Because of its potency both *in vitro* and *in vivo*, 5′pppRNA represents a specific and powerful trigger of innate immunity and a novel approach to antiviral therapy.

For the first time, an RNA-based agonist of RIG-I was shown to block the replication of multiple viruses; this broad-spectrum antiviral activity of 5′pppRNA is attributable in part to a potent stimulation of the inflammatory and antiviral response driven by the early induction of IRF, NF-κB, STAT, chemokines and pro-inflammatory cytokine genes. In parallel, we also observed an inhibition of genes involved in TGF-β signaling. Because of the immunosuppressive nature of the TGF-β pathway [42], inhibition of this transcriptional node could further potentiate immune activation in response to 5′pppRNA agonist. The emergence of apoptosis and ubiquitin signaling nodes at later times (24 h) suggests a role for cell death and ubiquitin-based signal modification in the antiviral response.

5′pppRNA stimulation of RIG-I triggered a complete IFN response. At 24 h post treatment, 5′pppRNA induced the expression of 97% of the genes stimulated by IFNα-2b treatment and the magnitude of ISG induction by 5′pppRNA was enhanced compared to IFNα-2b profile. Among the ISGs, the tripartite motif containing (TRIM) proteins, the IFITM proteins, MX1 and viperin exemplify the range of ISGs induced by RIG-I and all have been implicated as inhibitors of HIV-1, Influenza, VSV, West Nile, Dengue, and HCV [43]–[53]. In a recent study, high throughput screening of antiviral effectors identified a panel of broadly acting antiviral molecules, with the combined expression of multiple ISGs providing additive inhibitory effects against HCV replication [29]. Of the 28 validated antiviral ISGs identified, 19 were induced by 5′pppRNA in A549, including *IRF1*, *RIG-I*, *Mda5*, *IFITM3*.

The transcriptome analysis also identified a distinct subset of 968 genes specifically induced by 5′pppRNA - and not IFNα-2b - that additively or synergistically enhanced the antiviral response stimulated by 5′pppRNA treatment. Bioinformatics analysis identified a unique functional signature with up-regulated genes involved in inducing a wider range of signaling pathways and bridging innate and adaptive immune responses. The importance of genes uniquely induced by 5′pppRNA is highlighted by the antiviral response that limited influenza infection *in vitro* and *in vivo*, even in the absence of functional type I IFN signaling. We speculate that the extended range of genes induced by 5′pppRNA, compared to IFNα-2b, reflect the activation of multiple signaling pathways downstream of RIG-I/MAVS [10], versus the more-limited transactivation potential of the IFN-regulated JAK-STAT axis [26].

Type III IFNs (*IL29*, *IL28A*, *IL28B*) were among the most highly stimulated genes uniquely up-regulated in response to 5′pppRNA. Recent studies have demonstrated that both type I and type III IFNs activate similar components of the JAK-STAT pathways, although type III IFNs were shown to prolong the activation of JAK-STAT signaling and induce a delayed and stronger induction of ISGs, compared to type I IFNs [54]. IFNλs have been increasingly implicated in antiviral therapies: 1) IFNλ administration in mice stimulated expression of Mx1 and protected IFNαR−/− mice from lethal influenza challenge [55]; 2) IL29 blocked HIV-1 replication by inhibiting virus integration and post-transcriptional events [56]; and 3) the combination of IL29 and IFNα or IL29 and IFNγ enhanced the induction of antiviral genes and effectively inhibited HCV and VSV replication [57]. In addition, polymorphism in or near IFNλ3 gene correlated with spontaneous or treatment-induced clearance of Hepatitis C infection and IFNλ therapy is now actively investigated for the treatment of HCV [58]. Altogether these results indicate that specific induction of IFNλ by 5′pppRNA may contribute to immunity at the site of viral infection.

Of note, negative regulators of the innate immune response were also detected after 5′pppRNA stimulation. In addition to the induction of *SOCS1*, *USP18*, and *IFIT1* by both 5′pppRNA and IFNα-2b treatment, unique negative regulators activated exclusively by 5′pppRNA were also identified: *SOCS3* contributes to the inhibition of the JAK/STAT signaling [59], and hence limits the amplification of the IFN response; *A20* and *IκBα* inhibit the activation of the NFκB signaling complex [60], [61], which would prevent excessive inflammation. This observation suggests that targeting an upstream viral sensor may provide activation as well as negative feed-back regulation to terminate the immune response and prevent uncontrolled inflammation; as such, this approach may offer an advantage over IFN therapy in terms of limiting potential toxic side-effects.

Activation of the RIG-I signaling pathway using 5′pppRNA also induced an integrated set of genes and pathways that can efficiently bridge the innate and adaptive immune responses and utilize multiple arms of both systems. 5′pppRNA mobilized genes that enhance trafficking of immune cells such as neutrophils, monocytes, naïve and memory T cells and B cells, including *CCL17*, *CCL20*, *CXCL10*, *CCL3*, *CCL5* and many others. We also observed the induction of genes important for the activation of the effector arm of the adaptive immune system such as *IL-6*, which has been shown to enhance CD8+ T cell survival and killing potential [62]. These cytokines and chemokines certainly play a role in initiating the innate and adaptive immune cell response, which is critical for the generation of efficient immunity against multiple viral infections *in vivo*.

Intravenous administration of the RIG-I agonist stimulated a potent immune response *in vivo* that reached the lungs and prevented mortality associated with virus challenge. Histopathology analysis revealed diminished influenza-mediated lung damage and recruitment of inflammatory cells in infected lungs following 5′pppRNA treatment. The rapid control of virus replication, as demonstrated by significantly reduced virus titers in the lungs within 3 days of virus inoculation, may have prevented excessive immune cell recruitment early after infection. Influenza infection generates a complex pathogenesis mediated in part by viral- and immune-mediated damage [63]; therefore, the activation and recruitment of limited numbers of specific immune cell types, such as neutrophils, alveolar macrophages and dendritic cells may generate a beneficial antiviral microenvironment that additionally favor the initiation of adaptive immune response, which would eventually contribute to the *in vivo* efficacy of the agonist.

Other groups have recently reported that 5′pppRNA induces a protective RIG-I mediated antiviral response that inhibits the replication of influenza virus [64]–[66]. The inhibition of influenza infection by 5′pppRNA was dependent on the 5′ppp moiety and the secondary IFN response was crucial for mounting an effective antiviral response [64], [65]. Recently, a short RNA molecule with dual functionality was developed - a siRNA against influenza NP gene and an agonist of the RIG-I pathway [66]. This 5′pppRNA inhibited influenza infection *in vitro* and *in vivo*, but the contribution of RIG-I activation to the inhibition of influenza was not demonstrated. The defective interfering RNA produced during Sendai virus life cycle is the best characterized natural RIG-I ligand and is known to induce strong inflammatory response [67]. Interestingly, this molecule has adjuvant potential and could stimulate an antibody-dependent response directed to influenza antigens. Compared to these earlier studies, we adopted a systems approach to provide biochemical, transcriptional and biological mechanistic explanations for the antiviral efficacy observed *in vitro* and *in vivo*.

Activating natural host defense to prevent establishment and dissemination of viral infection is a valuable alternative strategy to antiviral drugs that specifically target viral processes. Interferon therapy has been used in the clinic for over two decades and has proven effective in the treatment of certain viral infections, mainly Hepatitis B and Hepatitis C [58], as well as malignancies and autoimmune diseases [68], [69]. However, IFN therapy is also associated with significant side effects that limit its use [70]. PolyI:C, another dsRNA immune modulator, is also being tested *in vitro* and *in vivo* and has demonstrated efficacy against respiratory infections [71]–[73]. Along with antiviral drugs, vaccination is the primary approach to reduce morbidity and mortality associated with viral infection. Increasing the immunogenicity of vaccines with molecular adjuvants eliciting cytokines, co-stimulatory molecules, or immunomodulatory factors enhance the vaccine-elicited immune responses. 5′pppRNA has the advantage of mimicking viral recognition to trigger an immune response analogous to natural viral infection. Furthermore, the response stimulated by 5′pppRNA was reminiscent of the integrated and multipotent response elicited early following immunization by the most protective vaccine identified so far, the yellow fever YD17 vaccine [74]. Therefore, an immune modulator such as a RIG-I agonist may not only function as an antiviral therapeutic, but may also serve as a vaccine adjuvant to increase the magnitude of the antiviral immune response elicited by vaccine epitopes. Thus, the present study not only demonstrated the prophylactic and therapeutic antiviral potential of 5′pppRNA, but also opens the door to further investigation of the potential of RIG-I agonists as vaccine adjuvants.

## Materials and Methods

### *In vitro* synthesis of 5′pppRNAs

The sequence of the 5′pppRNA was derived from the 5′ and 3′ UTRs of the VSV genome as previously described [17]. *In vitro* transcribed RNA was prepared using the Ambion MEGAscript T7 High Yield Transcription Kit according to the manufacturer′s instruction (Invitrogen, NY, USA). The template consisted of two complementary viral sequences containing T7 promoter that were annealed at 95°C for 5 minutes and cooled down gradually over night (5′-GAC GAA GAC AAA CAA ACC ATT ATT ATC ATT AAA ATT TTA TTT TTT ATC TGG TTT TGT GGT CTT CGT CTA TAG TGA GTC GTA TTA ATT TC-3′). The in vitro transcription reactions proceeded for 16 hours. 5′pppRNA was purified and isolated using the Qiagen miRNA Mini Kit (MD, USA). Homologous RNA without 5′ppp moiety was purchased from IDT (Integrated DNA Technologies Inc, Iowa, USA); dephosphorylation of the 5′pppRNA using CIAP (Invitrogen, NY, USA) generated identical results (data not shown). Secondary structure was predicted using the RNAfold WebServer (University of Vienna, Vienna, Austria). RNA was analysed on a denaturing 17% polyacrylamide, 7 M urea gel following digestion with 50 ng/ul of RNase A (Ambion, CA, USA) or 100 mU/ul of DNase I (Ambion, CA, USA) for 30 min.

### Cell culture, transfections, and luciferase assays

A549 were grown in F12K (Invitrogen, NY, USA) supplemented with 10% FBS and antibiotics. MEFs were grown in DMEM supplemented with 10% FBS, non-essential amino acids, and L-Glutamine (Wisent, Quebec, Canada). WT and RIG-I −/− MEFS were kind gifts from Dr. Shizuo Akira (Osaka University, Osaka, Japan) [75]. WT, Mda5−/−, TLR3−/−, TLR7−/− MEFS were kind gifts from Dr. Michael Diamond (Washington University, St Louis, USA) [76], [77]. Lipofectamine RNAiMax (Invitrogen, NY, USA) was used for transfections in A549 according to manufacturer's instructions. For luciferase assays, transfections were performed in wt and RIG-I−/−; wt and Mda5−/−, TLR3−/−, TLR7−/− MEFs using Lipofectamine 2000 (Invitrogen, New York, USA) or jetPRIME (PolyPlus, France), respectively. Plasmids encoding GFP-ΔRIG-I, IRF-7, pRLTK, IFNα4/pGL3 and IFNβ/pGL3 were previously described [78]. MEFs were co-transfected with 200 ng pRLTK reporter (Renilla luciferase for internal control), 200 ng of reporter gene constructs, together with 5′pppRNA (500 ng/ml) or 100 ng of a plasmid encoding a constitutively active form of RIG-I (ΔRIG-I) [41]. IRF7 plasmid (100 ng) was added for transactivation of IFNα4 promoter. At 24 h after transfection, reporter gene activity was measured by Dual-Luciferase Reporter Assay, according to manufacturer's instructions (Promega, Wisconsin, USA). Relative luciferase activity was measured as fold induction (relative to the basal level of reporter gene). For siRNA knock down, A549 cells were transfected with 50 nM (30 pmol) of human RIG-I (sc-61480), IFN-α/βR α (sc-35637) and β (sc-40091) chain, or control siRNA (sc-37007) (Santa Cruz Biotechnologies, Dallas, USA) using Lipofectamine RNAi Max (Invitrogen, NY, USA) according to the manufacturer's guidelines. Treatment with 5′pppRNA was performed 48 hrs later.

### Immunoblot analyses

Whole cell extracts were separated in 8% acrylamide gel by SDS-PAGE and were transferred to a nitrocellulose membrane (BioRad, Mississauga, Canada) at 4°C for 1 h at 100 V in a buffer containing 30 mM Tris, 200 mM glycine and 20% methanol. Membranes were blocked for 1 h at room temperature in 5% dried milk (wt/vol) in PBS and 0.1% Tween-20 (vol/vol) and then were probed with primary antibodies: anti-pIRF3 at Ser396 (EMD Millipore, Massachusetts, USA), anti-IRF3 (IBL, Japan), anti-RIG-I (EMD Millipore, Massachusetts, USA), anti-ISG56 (Thermo Fischer Scientific, Massachusetts, USA), anti-pSTAT1 at Tyr701 (Cell Signaling Technology, Inc, Massachusetts, USA), anti-STAT1 (Santa Cruz Biotechnology), anti-NS1(Santa Cruz Biotechnology), anti-pIkBα at Ser32 (Cell Signaling Technology, Inc, Massachusetts, USA), anti-IκBα (Cell Signaling Technology, Inc, Massachusetts, USA), anti-NOXA (EMD Millipore, Massachusetts, USA), anti-cleaved Caspase 3 (Cell Signaling Technology, Inc, Massachusetts, USA), anti-PARP (Cell Signaling Technology, Inc, Massachusetts, USA), anti-β-Actin (EMD Millipore, Massachusetts, USA). Antibody signals were detected by chemiluminescence using secondary antibodies conjugated to horseradish peroxidise and an ECL detection kit (Amersham Biosciences, Inc, NJ, USA)

### IRF3 dimerization

Whole cell extracts were prepared in NP-40 lysis buffer (50 mM Tris, pH 7.4, 150 mM NaCl, 30 mM NaF, 5 mM EDTA, 10% glycerol, 1.0 mM Na_3_VO_4_, 40 mM β-glycerophosphate, 0.1 mM phenylmethylsulfonyl fluoride, 5 µg/ml of each leupeptin, pepstatin, and aproptinin, and 1% Nonidet P-40). WCE was then subjected to electrophoresis on 7.5% native acrylamide gel, which was pre-run for 30 min at 4°C. The electrophoresis buffers were composed of an upper chamber buffer (25 mM Tris, pH 8.4, 192 mM glycine, and 1% sodium deoxycholate) and a lower chamber buffer (25 mM Tris, pH 8.4, 192 mM glcine). Gels were soaked in SDS running buffer (25 mM Tris, pH 8.4, 192 mM glycine, 0.1% SDS) for 30 min at 25°C and were then transferred to nitrocellulose membrane (Amersham Biosciences). Membranes were blocked in PBS containing 5% milk (wt/vol) and 0.05% Tween-20 (vol/vol) for 1 h at 25°C and blotted with an antibody against IRF3 (IBL, Japan). Antibody signals were detected by chemiluminescence using secondary antibodies conjugated to horseradish peroxidise and an ECL detection kit (Amersham Biosciences, Inc, NJ, USA)

### ELISA

The release of human IFNα (multiple subunits) and IFNβ in culture supernatants of A549, and murine IFNα and IFNβ in serum or lung homogenate (20% w/v) from mice in response to 5′pppRNA were measured by ELISA according to manufacturer's instructions (PBL Biomedical Laboratories, Piscataway, NJ).

### Primary cell isolation

PBMCs were isolated from freshly collected blood using a Lymphocyte Separation Medium (Cellgro) as per manufacturer's instructions. After isolation, total PBMCs were frozen in heat-inactivated FBS with 10% DMSO. On experimental days, PBMCs were thawed, washed and placed at 37°C for 1 hr in RPMI with 10% FBS supplemented with Benzonaze™ nuclease (Novagen) to prevent cell clumping. The optimal concentration of 5′pppRNA to efficiently activate PBMC with minimal cytotoxicity was 100 ng/mL (data not shown). PBMCs were isolated from the blood of patients in a study both approved by IRB and by the VGTI Florida Institutional Biosafety Committee (2011-6-JH-1). Written informed consent approved by the Vaccine and Gene Therapy Institute Florida Inc. ethics review board (FWA#161) was provided and signed by study participants. Research was conformed to ethical guidelines established by the ethics committee of the OHSU VGTI and Martin Health System.

### Virus production and infection

VSV-GFP, which harbors the methionine 51 deletion in the matrix protein-coding sequence [79], was kindly provided by J. Bell (Ottawa Health Research Institute, CA). Virus stock was grown in Vero cells, concentrated from cell-free supernatants by centrifugation, and titrated by standard plaque assay as described previously [80]. The recombinant vaccinia-GFP virus (VVΔE3L-REV), a revertant strain of the E3L deletion mutant, was kindly provided by Jingxin Cao (National Microbiology Laboratory, Public Health Agency of Canada, Winnipeg) [81], [82]. Dengue virus serotype 2 (DENV-2) strain New Guinea C was grown in C6/36 insect cells for 7 days. Briefly, cells were infected at a MOI of 0.5, and 7 days after infection, cell supernatants were collected, clarified and stored at −80°C. Titers of DENV stocks were determined by serial dilution on Vero cells, with intracellular immunofluorescent staining of DENV E protein at 24 h post-infection and denoted as infectious units per ml. Titers of Dengue virions were determined by standard plaque assay in Vero cells; plaques were fixed, stained and counted 5 days later. In infection experiments, both PBMCs and A549 cells were infected in a small volume of medium without FBS for 1 hour at 37°C and then incubated with complete medium for 24 h prior to analysis.

HIV-GFP virus is an NL4-3 based virus designed to co-express Nef and eGFP from a single bicistronic RNA. HIV-GFP particles were produced by transient transfection of pBR43IeG-nef+ plasmid into 293T cells as described previously [83], [84]. Briefly, 293T cells were transfected with 22.5 µg of pBR43IeG-nef+ plasmid by polyethylenimine precipitation. Media was replaced 14–16 h post-transfection, and viral supernatants were harvested 48 hrs later, cleared by low-speed centrifugation and filtered through a 0.45 µm low binding protein filter. High-titer viral stocks were prepared by concentrating viral supernatants 100-fold through filtration columns (Amicon), then aliquoted and stored at −80°C. Viral titers were determined by p24 level (ELISA) and TCID50; briefly, 10-fold serial dilutions of concentrated viral supernatants were used to infect PBMCs from two donors pre-activated for 3 days with 10 µg/ml of PHA. Half of the media was replaced on day 4, and 7 days after infection, supernatants were harvested and processed for p24 by ELISA. The Reed–Muench method was used to calculate the TCID50. For HIV infection, CD14+ monocytes were negatively selected using the EasySep Human Monocytes Enrichment Kit (Stem Cell) as per manufacturer's instructions. Isolated cells were transfected with 5′pppRNA (100 ng/ml) using Lyovec (Invitrogen) according to the manufacturer's protocol. Supernatants were harvested 24 h after stimulation and briefly centrifuged to remove cell debris. CD4+ T cells were isolated using EasySep™ Human CD4+ T cells Enrichment Kit (Stem Cell) according to the manufacturer's guidelines. Purified CD14+ monocytes and CD4+ T cells were allowed to recover 1 h in RPMI containing 10% FBS at 37°C with 5% CO_2_ before experiments. For HIV infection, anti-CD3 Ab (0.5 µg/ml) were immobilized for 2 hours in 24-well plate and CD4+ T cells were then added along with anti-CD28 Ab (1 µg/ml) to allow activation of T cells for 2 days. After activation, cells were incubated for 4 hours with supernatant of 5′pppRNA-stimulated monocytes and infected with HIV-GFP at an MOI of 0.1. Supernatant from the monocytes was left for another 4 h before adding complete medium.

HCV RNA was synthesized using the Ambion MEGAscript T7 High Yield Transcription Kit using linearized pJFH1 DNA (a generous gift Takaji Wakita; National Institute of Infectious Diseases, Shinjuku-ku) as template. Huh7 cells were electroporated with 10 mg of HCV RNA and at 5 days post-transfection, virus containing supernatant was collected, filtered (0.45 µm) and stored at −80°C (HCVcc). Huh7 or Huh7.5 cells were pre-treated with 5′pppRNA (10 ng/mL) for 24 h. Supernatants containing soluble factors induced following 5′pppRNA treatment was removed and kept aside during infection. Cells were washed once with PBS and infected with 0.5 ml of undiluted HCVcc for 4 h at 37°C; then, supernatant was added back. At 48 h post-infection, WCEs were prepared; expression of HCV NS3 protein was detected by Western blot (Abcam, Toronto, Ca)

Influenza H1N1 strain A/Puerto Rico/8/34 was kindly provided by Veronika von Messling (Duke-NUS, Singapore). Viral stock was amplified in Madin-Darby canine kidney (MDCK) cells and virus titer was determined by standard plaque assay [85]. Cells were infected in 1 ml medium without FBS for 1 hour at 37°C. Inoculum was aspirated and cells were incubated with complete medium for 24 hours, prior to analysis. For viral infections, supernatants containing soluble factors induced following 5′pppRNA treatment was removed and kept aside during infection. Cells were washed once with PBS and infected in a small volume of medium without FBS for 1 h at 37°C; then supernatant was added back for the indicated period of time.

### Flow cytometry analyses

The percentage of cells infected with VSV, Vaccinia and HIV was determined based on GFP expression. The percentage of cells infected with Dengue was determined by standard intra-cellular staining. Cells were stained with a mouse IgG2a mAb specific for DENV-E-protein (clone 4G2) followed by staining with a secondary anti-mouse antibody coupled to PE (Jackson Immuno Research). PBMCs infected with DENV were first stained with anti-human CD14 Alexa Fluor 700 Ab (BD Biosciences). Cells were analyzed on a LSRII flow cytometer (Becton Dickinson). Compensation calculations and cell population analysis were done using FACS Diva.

### In vivo administration of 5′pppRNA and influenza infection model

C57Bl/6 mice (8 weeks-old) were obtained from Charles River Laboratories. MAVS−/− and WT (mixed 129/SvEv-C57Bl/6 background) were obtained from Z. Chen (The Howard Hughes Medical Institute, US). TLR3−/− mice were obtained from Taconic. IFNα/βR−/− mice were bred on a C57Bl/6 background. For intra-cellular delivery, 25 ug of 5′pppRNA was complexed with in vivo-jetPEI (PolyPlus, France) at an N/P ratio of 8 as per manufacturer's instructions and administered intravenously via tail vein injection. Unless otherwise indicated, 5′pppRNA was administered on the day prior to infection (Day −1) and on the day of infection (Day 0). Mice under 4% isoflurane anesthesia were infected intra-nasally with 500 PFU of Influenza A/PR/8/34 (Day 0). For viral titers, lungs were homogenized (20% wt/vol) in DMEM and titers were determined by standard plaque assay as previously described [85]. Briefly, confluent Madin-Darby Canine Kidney Cells (MDCK) were incubated with 250 µL of serial Log10 dilutions for 30 minutes, the sample was aspirated, and cells overlaid with 3 ml of 1.6% agarose in DMEM. Plaques were fixed and counted 48 h later. All animal experimentations were performed according to the guidelines of the Canadian Council on Animal Care and approved by the McGill University Animal Care Committee. The IFNα/βR−/− animal experimentations were approved by the INRS Institutional Animal Care and Use Committee.

### Histology and histopathology

All five lobes of the lungs were collected and fixed in 10% neutral-buffered formalin for 24 h. The organs were paraffin-embedded and 4 µm sections were cut and stained with hematoxyline and eosin staining (H&E). The slides were analysed by a board-certified independent veterinary pathologist.

### Microarray analysis

The kinetics and the comparison to IFNα-2b were performed as two separate experiments. A549 cells were stimulated with either 5′pppRNA (10 ng/ml) or IFNα-2b (100 IU/ml or 1000 IU/ml) for designated times. IFNα-2b (Intron A) was purchased from Schering Plough (Kenilworth, NJ). Cells were collected and lysed for RNA extraction (Qiagen, Valencia, CA, USA). Reverse transcription reactions were performed to obtain cDNAs which were hybridized to the Illumina Human HT-12 version 4 Expression BeadChip according to the manufacturer's instruction, and quantified using an Illumina iScan System. The data were collected with Illumina GenomeStudio software. First, arrays displaying unusually low median intensity, low variability, or low correlation relative to the bulk of the arrays were discarded from the rest of the analysis. Quantile normalization, followed by a log2 transformation using the Bioconductor package LIMMA was applied to process microarrays. To account for variability between batches, the data were adjusted using the ComBat procedure (http://dx.doi.org/10.1093/biostatistics/kxj037). Missing values were imputed with the R package (http://cran.r-project.org/web/packages/impute/index.html). In order to identify differentially expressed genes between treated and controls (untreated) samples, the LIMMA package [86] from Bioconductor was used. For data mining and functional analyses, genes that satisfied a *p*-value (<0.001) with ≥2 fold change (up or down) were selected. Probes that do not map to annotated RefSeq genes and control probes were removed. The expected proportions of false positives (FDR) were estimated from the unadjusted *p*-value using the Benjamini and Hochberg method [87]. All network analysis was done with Ingenuity Pathway Analysis (IPA: Ingenuity systems, Redwood City, CA). The differentially expressed genes selected based on above criteria were mapped to the ingenuity pathway knowledge base with different colors (red: up-regulated; green: down-regulated). The significance of the association between the dataset and the canonical pathway was measured in two ways: (1) A ratio of the number of genes from the dataset that map to the pathway divided by the total number of genes that map to the canonical pathway was displayed; (2) by over-representation analysis Fisher's exact test was used to calculate a *p*-value determining the probability that the association between the genes in the dataset and the canonical pathway is explained by chance alone. The pathways were ranked with −log *p*-values. Microarray data have been deposited in the NCBI Gene Expression Omnibus.

### Quantitative real-time PCR

Total RNA was isolated from cells using RNeasy Kit (Qiagen, Valencia, CA, USA). Spleen and lungs were homogenized in RLT buffer and RNA isolated as per manufacturer's instruction. 1 ug of RNA was reverse transcribed using High-Capacity cDNA Reverse Transcription Kits from Applied Biosystems according to manufacturer's instructions. Parallel reactions without reverse transcriptase were included as negative controls. Relative amount of an intracellular RNA of interest was quantified by real-time PCR on a 7500 fast real-time PCR system and expressed as a fold change using SYBR Green (Roche) according to the manufacture's protocol. All data presented are relative quantification with efficiency correction based on the relative expression of target genes versus GAPDH as reference gene. Primers sets used for these studies are presented in Table S1.

## Supporting Information

Figure S1 **Quantitative real-time RT-PCR validation of genes induced by 5′pppRNA treatment.** Quantitative real-time RT-PCR was performed to validate the kinetic observed in the microarray for selected genes. A549 cells were transfected with 10 ng/ml of 5′pppRNA using Lipofectamine RNAiMax for designated periods of time. RNA was extracted from time-course samples and subjected to real-time RT-PCR analysis. Results are from a representative experiment; error bars represent SEM of three independent samples.
(TIF)

Figure S2 **Transcriptional profiling following 5′pppRNA stimulation of A549 cells.** (**A**) Left panel: Temporal analysis of up-regulated genes at 6 h (FC≥2 at 6 h). Right panel: Temporal analysis of genes that are exclusively induced significantly at 24 h (FC≤2 at 6 h and FC≥2 at 24 h). Genes with highest fold change under these conditions are listed. (**B**) Node analyses of transcriptome activation 6 h (left panel) and 24 h (right panel) post 10 ng/ml 5′pppRNA stimulation. Ingenuity Pathway Analysis software was used to generate transcriptome signalling networks centered around IRF1 and IRF7. Genes coloured in red were up-regulated, in green were down-regulated. (**C**) Node analyses of transcriptome activation 24 h post stimulation with either 10 ng/ml 5′pppRNA (left panel) or 1000 IU/ml IFNα-2b (right panel). Ingenuity Pathway Analysis Software was used to generate signaling networks surrounding IRF7 and NF-κB. Genes in red were up-regulated (FC≥2.0), in green were down-regulated (FC≤2.0).
(TIF)

Figure S3 **Quantification of VSV and Dengue virions.** (**A**) Left panel: VSV virus titer from the supernatants from the experiment described in Fig. 6A was determined by standard plaque assay. Right panel: WCEs were analysed by western blotting for VSV protein expression. (**B**) Left panel: Dengue virus titer from the supernatants from the experiment described in Fig. 6A was determined by standard plaque assay. Right panel: RNA was extracted and analysed by real-time RT-PCR using primers specific for Dengue RNA (Forward 5′ CAA GGC GAG ATG AAG CTG TA 3′; Reverse 5′ GGT CTT TCC CAG CGT CAA TA 3′).
(TIF)

Figure S4 **Stimulation of an immune response by 5′pppRNA** ***in vivo*** **.** C57Bl/6 mice were injected intravenously with 25 ug of 5′pppRNA in complex with In vivo Jet-PEI or non-treated (NT, 0 h). (**A**) Serum (left panels) and lung homogenate (20% w/v in PBS; right panels) were collected at the indicated time point and ELISA for IFNβ (top panels) and IFNα (bottom panels) were performed. Error bars represent SEM from three different animals. (**B**) Spleen (left panels) and lungs (right panels) were collected at the indicated time point. RNA was extracted subjected to real-time RT-PCR. Error bars represent SEM from three different animals. (**C**) Lungs were collected at 6 h and 24 h post 5′pppRNA administration. Lungs were minced and digested with collagenase IV and DNase I for 30 minutes, with vigorous mixing after 15 minutes, and then filtered through a 70 µm nylon filter. Cells were analysed by flow cytometry and the proportion of cells relative to CD45+ leukocyte is reported. Error bars represent SEM from four different animals.
(TIF)

Table S1 **Primer sequences for real-time RT-PCR.** List of forward and reverse primers used for real-time RT-PCR.
(DOC)
