# Correction: Systems Analysis of a RIG-I Agonist Inducing Broad Spectrum Inhibition of Virus Infectivity

**DOI:** 10.1371/annotation/8fa70b21-32e7-4ed3-b397-ab776b5bbf30

**Published:** 2013-08-08

**Authors:** Marie-Line Goulet, David Olagnier, Zhengyun Xu, Suzanne Paz, S. Mehdi Belgnaoui, Erin I. Lafferty, Valérie Janelle, Meztli Arguello, Marilene Paquet, Zhong He, Khader Ghneim, Stephanie Richards, Andrew Smith, Peter Wilkinson, Mark Cameron, Ulrich Kalinke, Salman Qureshi, Alain Lamarre, Elias K. Haddad, Rafick Pierre Sekaly, Suraj Peri, Siddharth Balachandran, Rongtuan Lin, John Hiscott

Zhong He was not included in the author byline. He should be listed as the tenth author and affiliated with the Division of Infectious Diseases, Vaccine & Gene Therapy Institute of Florida, Port Saint Lucie, Florida, USA. The contributions of this author are as follows: Performed the experiments and Analyzed the data.

