# Supplementary figures and images for: Systems Analysis of a RIG-I Agonist Inducing Broad Spectrum Inhibition of Virus Infectivity
Source: PLoS Pathog. 2013 Apr 25;9(4):e1003298. doi: 10.1371/journal.ppat.1003298 (PMC3635991; doi:10.1371/journal.ppat.1003298)

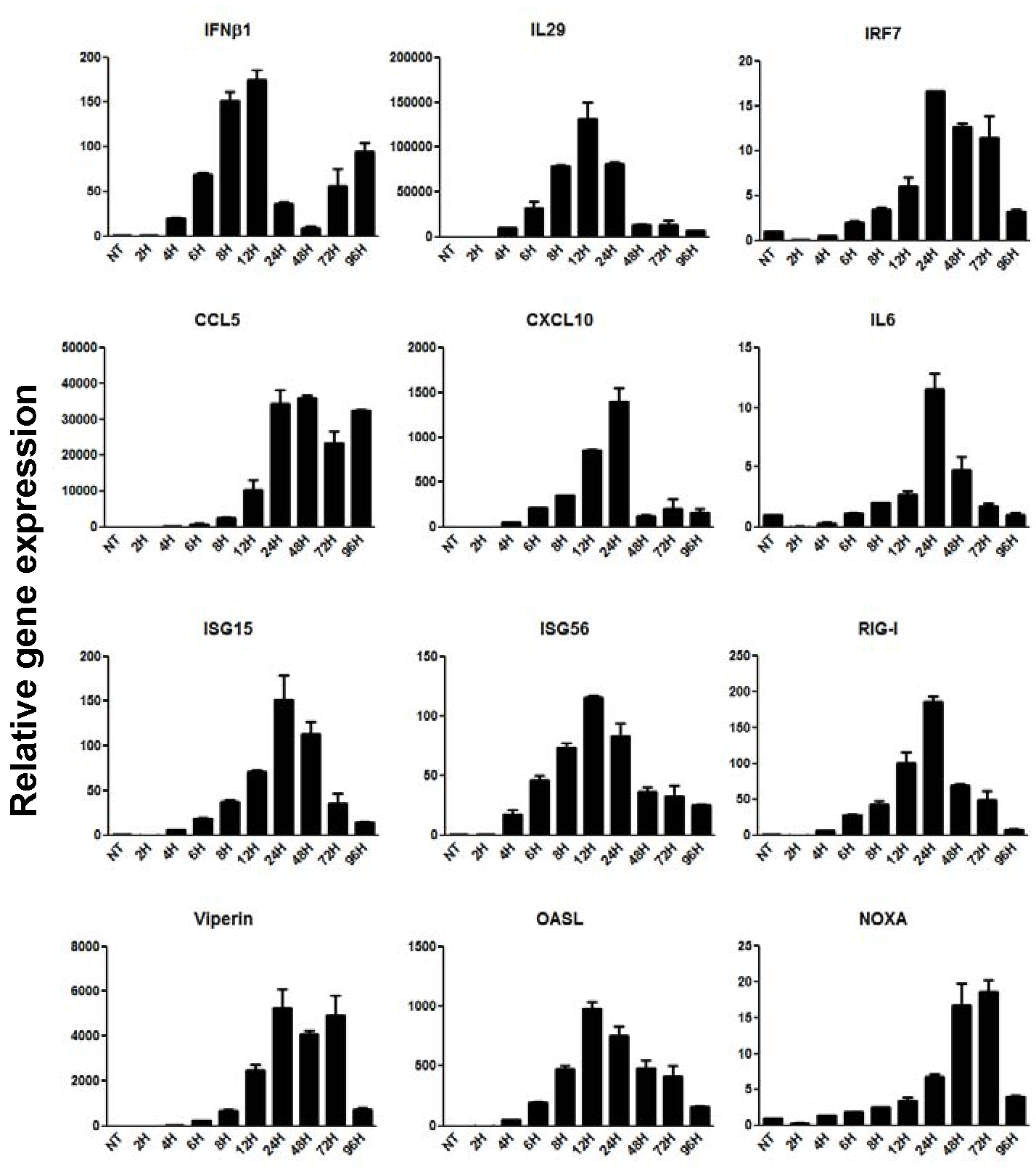

Supplement: Figure S1 — Quantitative real-time RT-PCR validation of genes induced by 5′pppRNA treatment. Quantitative real-time RT-PCR was performed to validate the kinetic observed in the microarray for selected genes. A549 cells were transfected with 10 ng/ml of 5′pppRNA using Lipofectamine RNAiMax for designated periods of time. RNA was extracted from time-course samples and subjected to real-time RT-PCR analysis. Results are from a representative experiment; error bars represent SEM of three independent samples. (TIF) [file ppat.1003298.s001.tif]

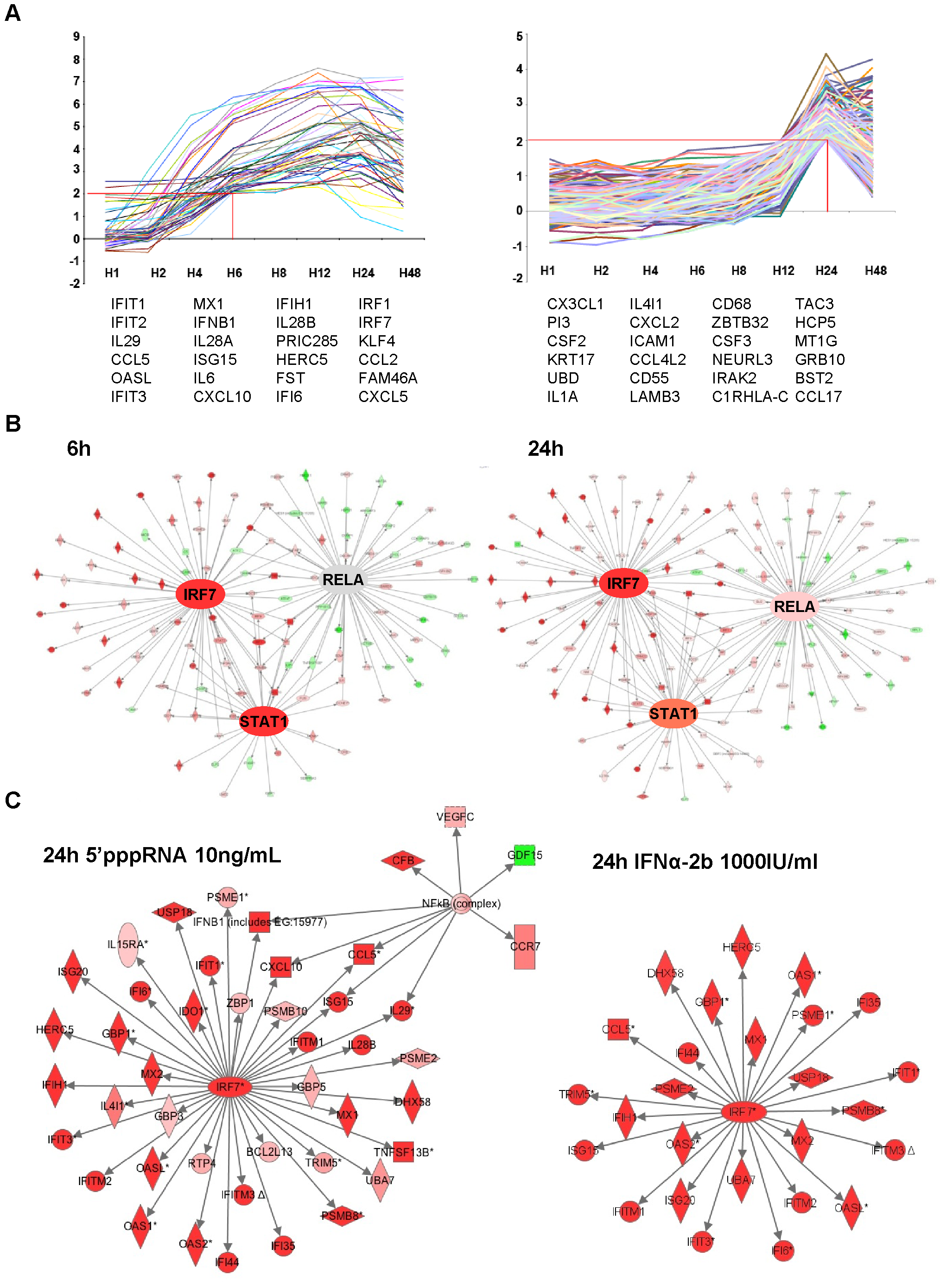

Supplement: Figure S2 — Transcriptional profiling following 5′pppRNA stimulation of A549 cells. (A) Left panel: Temporal analysis of up-regulated genes at 6 h (FC≥2 at 6 h). Right panel: Temporal analysis of genes that are exclusively induced significantly at 24 h (FC≤2 at 6 h and FC≥2 at 24 h). Genes with highest fold change under these conditions are listed. (B) Node analyses of transcriptome activation 6 h (left panel) and 24 h (right panel) post 10 ng/ml 5′pppRNA stimulation. Ingenuity Pathway Analysis software was used to generate transcriptome signalling networks centered around IRF1 and IRF7. Genes coloured in red were up-regulated, in green were down-regulated. (C) Node analyses of transcriptome activation 24 h post stimulation with either 10 ng/ml 5′pppRNA (left panel) or 1000 IU/ml IFNα-2b (right panel). Ingenuity Pathway Analysis Software was used to generate signaling networks surrounding IRF7 and NF-κB. Genes in red were up-regulated (FC≥2.0), in green were down-regulated (FC≤2.0). (TIF) [file ppat.1003298.s002.tif]

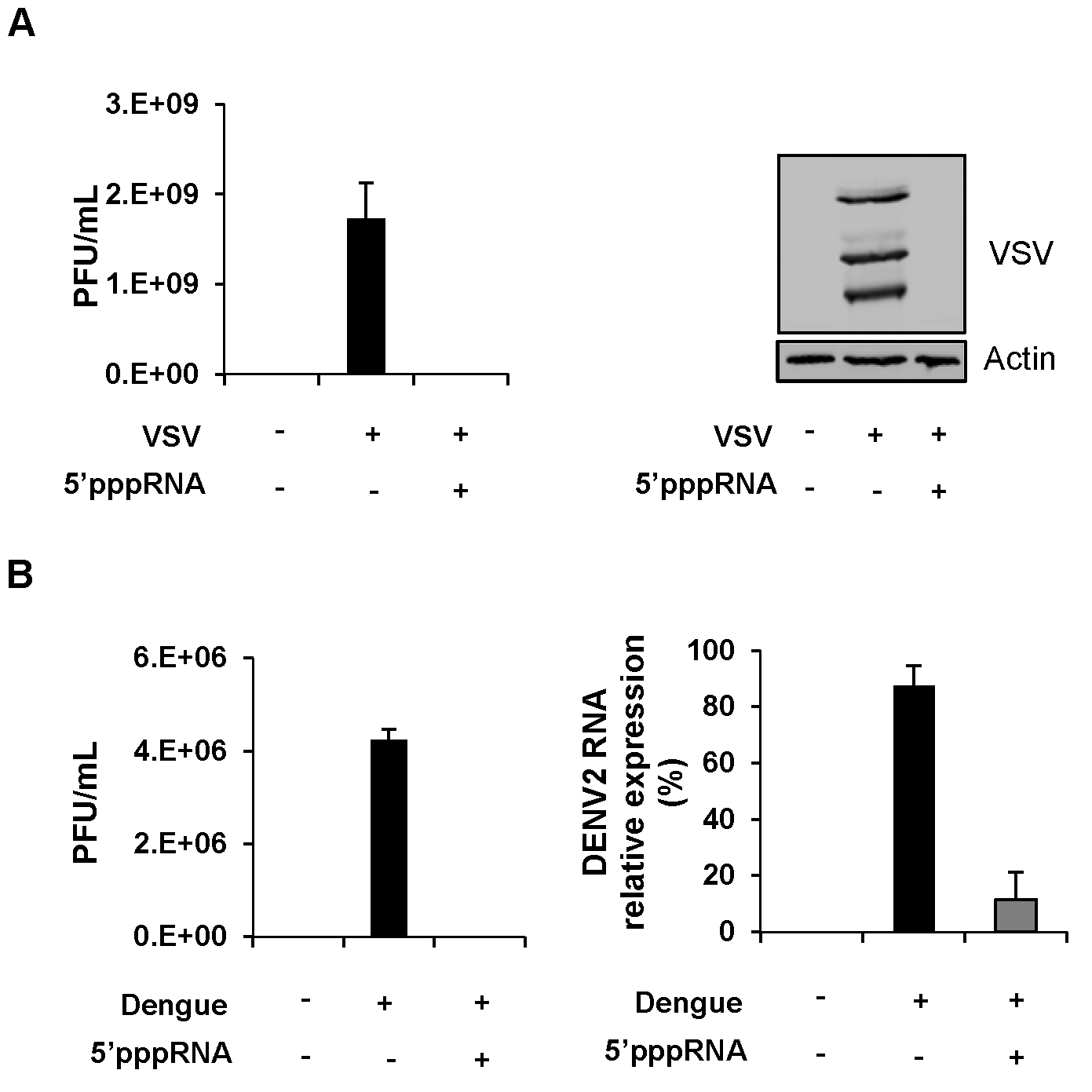

Supplement: Figure S3 — Quantification of VSV and Dengue virions. (A) Left panel: VSV virus titer from the supernatants from the experiment described in Fig. 6A was determined by standard plaque assay. Right panel: WCEs were analysed by western blotting for VSV protein expression. (B) Left panel: Dengue virus titer from the supernatants from the experiment described in Fig. 6A was determined by standard plaque assay. Right panel: RNA was extracted and analysed by real-time RT-PCR using primers specific for Dengue RNA (Forward 5′ CAA GGC GAG ATG AAG CTG TA 3′; Reverse 5′ GGT CTT TCC CAG CGT CAA TA 3′). (TIF) [file ppat.1003298.s003.tif]

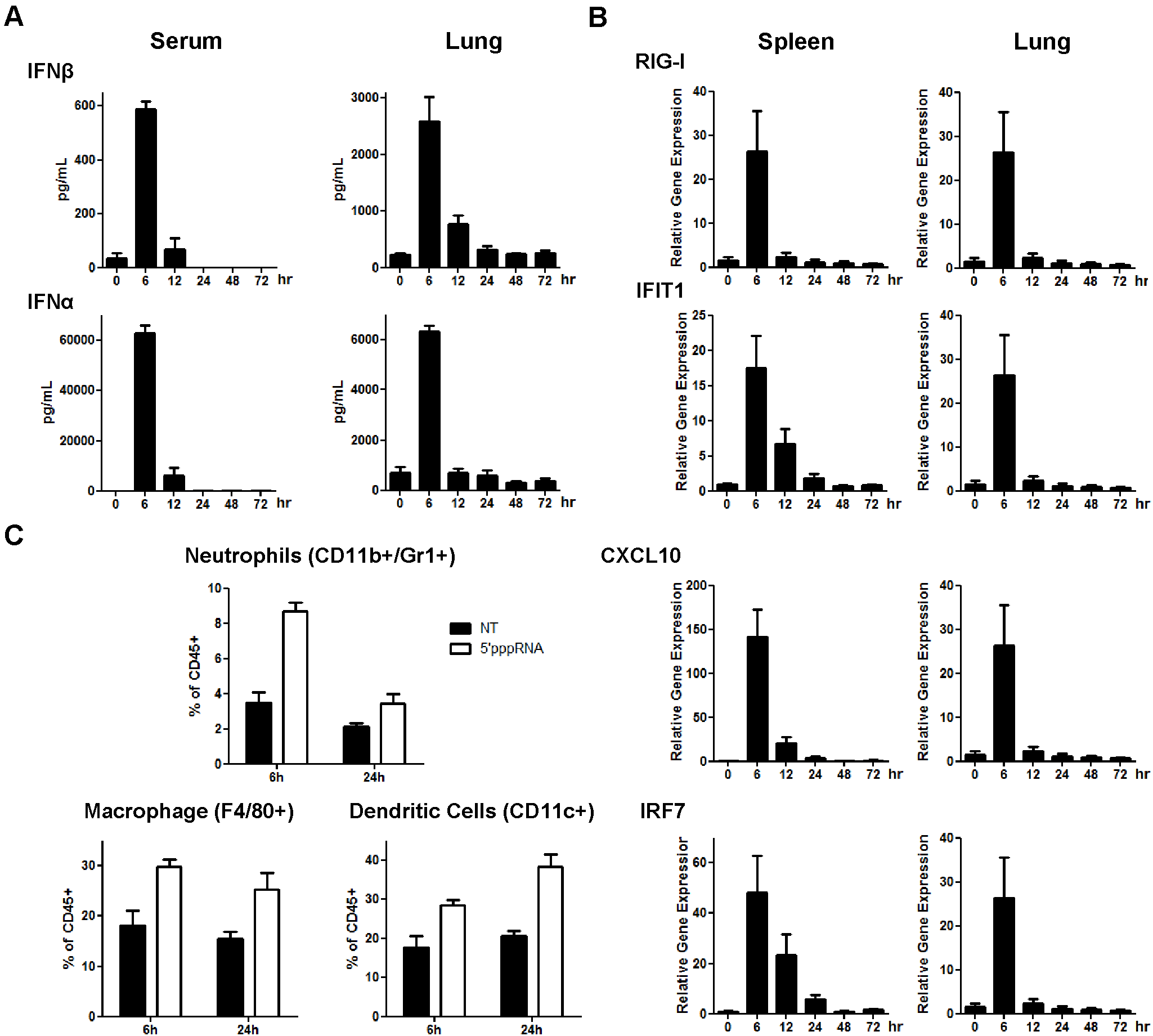

Supplement: Figure S4 — Stimulation of an immune response by 5′pppRNA in vivo . C57Bl/6 mice were injected intravenously with 25 ug of 5′pppRNA in complex with In vivo Jet-PEI or non-treated (NT, 0 h). (A) Serum (left panels) and lung homogenate (20% w/v in PBS; right panels) were collected at the indicated time point and ELISA for IFNβ (top panels) and IFNα (bottom panels) were performed. Error bars represent SEM from three different animals. (B) Spleen (left panels) and lungs (right panels) were collected at the indicated time point. RNA was extracted subjected to real-time RT-PCR. Error bars represent SEM from three different animals. (C) Lungs were collected at 6 h and 24 h post 5′pppRNA administration. Lungs were minced and digested with collagenase IV and DNase I for 30 minutes, with vigorous mixing after 15 minutes, and then filtered through a 70 µm nylon filter. Cells were analysed by flow cytometry and the proportion of cells relative to CD45+ leukocyte is reported. Error bars represent SEM from four different animals. (TIF) [file ppat.1003298.s004.tif]
