# Supplementary material for: Systems Analysis of a RIG-I Agonist Inducing Broad Spectrum Inhibition of Virus Infectivity
Source: PLoS Pathog. 2013 Apr 25;9(4):e1003298. doi: 10.1371/journal.ppat.1003298 (PMC3635991; doi:10.1371/journal.ppat.1003298)
Supplement: Table S1 — Primer sequences for real-time RT-PCR. List of forward and reverse primers used for real-time RT-PCR. (DOC) [file ppat.1003298.s005.doc]

**Table S1: Primer sequences for real-time RT-PCR**

| **Gene** | **Primer sequence** |
| --- | --- |
| **Human** |  |
| IFNB1 forward | 5’TTGTGCTTCTCCACTACAGC3’ |
| IFNB1 reverse | 5’CTGTAAGTCTGTTAATGAAG3’ |
| IL29 forward | 5’GGACGCCTTGGAAGAGTCACT3’ |
| IL29 reverse | 5’AGAAGCCTCAGGTCCCAATTC3’ |
| IRF7 forward | 5’CTTCGTGATGCTGCGAGATA3’ |
| IRF7 reverse | 5’AAGCCCTTCTTGTCCCTCTC3’ |
| CCL5 forward | 5’CTTTFTCACCCGAAAGAACC3’ |
| CCL5 reverse | 5’CTGTAAGTCTGTTAATGAAG3’ |
| CXCL10 forward | 5’TCTTCTCACCCTTCTTTTTCATTGT3’ |
| CXCL10 reverse | 5’TTCCTGCAAGCCAATTTTGTC3’ |
| IL6 forward | 5’GGAGACTTCCTGGTGAAAA3’ |
| IL6 reverse | 5’ATCTGAGGTGCCCATGCTAC3’ |
| ISG15 forward | 5’AGCTCCATGTCGGTGTCAG3’ |
| ISG15 reverse | 5’GAAGGTCAGCCAGAACAGGT3’ |
| ISG56 forward | 5’CAACCAAGCAAATGTGAGGA3’ |
| ISG56 reverse | 5’AGGGGAAGCAAAGAAAATGG3’ |
| RIG-I forward | 5’GCAGAGGCCGGCATGAC3’ |
| RIG-I reverse | 5’AATCCCATCACCATCTTCCA3’ |
| Viperine forward | 5’CACAAAGAAGTGTCCTGCTTGGT3’ |
| Viperine reverse | 5’AAGCGCATATATTTCATCCAGAATAAG3’ |
| OASL forward | 5’GGATCTTCTCCCACACTCACATCT3’ |
| OASL reverse | 5’CACCATCAGGATTCTTCACGAA3’ |
| NOXA forward | 5’AGCTGGAAGTCGAGTGTGCT3’ |
| NOXA reverse | 5’TCCTGAGCAGAAGAGTTTGGA3’ |
| GAPDH forward | 5’AATCCCATCACCATCTTCCA3’ |
| GAPDH reverse | 5’TGAGTCCTTCCACGATACCA3’ |
| **Murine** |  |
| RIG-I forward | AAGCAAGGCTGATGAGGATG |
| RIG-I reverse | CTCGCAATGTTGTACCCAAG |
| IFIT1 forward | CTCTGAAAGTGGAGCCAGAAAAC |
| IFIT1 reverse | AAATCTTGGCGATAGGCTACGA |
| CXCL10 forward | AAGTGCTGCCGTCATTTTCT |
| CXCL10 reverse | CACTGGGTAAAGGGGAGTGA |
| IRF7 forward | AAGCATTTTCGGTCGTAGGG |
| IRF7 reverse | GAGCCCAGCATTCTCTTG |
| GAPDH forward | AGGAGCGAGACCCCACTAAC |
| GAPDH reverse | GTGGTTCACACCCATCACAA |
